# Fast Hydrogel Micro‐Actuators Driven by Electric Fields

**DOI:** 10.1002/adma.74787

**Published:** 2026-09-05

**Authors:** Annaël Sort‐Montenegro, Jason M. Delente, Žiga Roblek, Yekaterina Tskhe, Luke Dowling, Colm Delaney, Larisa Florea

**Affiliations:** ^1^ School of Chemistry & AMBER The SFI Research Centre for Advanced Materials and BioEngineering Research Trinity College Dublin Dublin Ireland; ^2^ Department of Mechanical & Manufacturing Engineering Trinity College Dublin Dublin Ireland

**Keywords:** direct laser writing, electro‐active hydrogels, microfabrication, soft actuators, two‐photon polymerization

## Abstract

Electrically‐driven hydrogels are crosslinked, charged polymer networks that can deform in an electric field, prompted by osmotic pressure changes. To date, such actuators generally suffer from sluggish response time, with equilibrium actuation times ranging from minutes to hours in aqueous electrolytes, and lack sophisticated design, due to established manufacturing protocols. Herein, these limitations are overcome through the fabrication of polyelectrolyte hydrogel microstructures via two‐photon polymerization (2PP). This approach allows for the realization of microscale electrically‐driven actuators exhibiting fast actuation (∼200 ms equilibrium time). The work highlights three photoresist formulations for poly(anionic) and poly(cationic) hydrogel networks and their fabrication via 2PP to produce micro‐electro‐actuators with sub‐micron features. The electrically‐driven actuation performance is investigated by varying the hydrogel composition, actuator geometry, along with electric field strength and direction, and local environment (pH and electrolyte concentration) during actuation. It was determined that micro‐cantilevers of 80 × 20 × 10 µm^3^ reached equilibrium bending of up to 44.9 ± 7.8°, in ∼ 200 ms, in response to electric fields of 6 V mm^−1^. This pioneering work marks the first integration of 2PP with electrically actuated gelatin‐based hydrogels, showcasing micro‐electro‐actuators with rapid and programmable 4D motion.

## Graphical abstract

We report the fabrication of chemically programmable electroactuating microstructures through the first integration of two‐photon polymerization with electrically actuated gelatin methacrylate‐based hydrogels. By tailoring the charge of the polymer network, we demonstrate programmable polarity‐dependent bending, enabling microstructures that selectively bend toward either the anode or cathode under identical stimulation conditions.

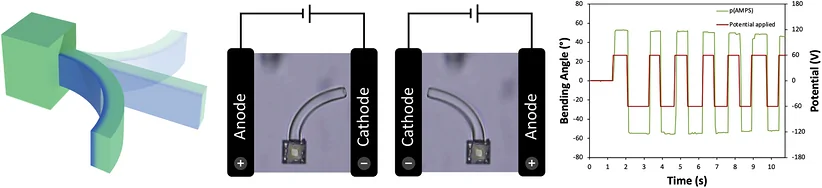

## 1 Introduction

Electro‐active polymers (EAPs) have attracted great interest over the years owing to their ability to translate an electrical stimulus into a mechanical response [1, 2]. To date, they have found application in numerous fields, including tissue engineering [3, 4], drug delivery [5, 6], sensors and actuators [2, 7, 8], lenses [7, 9], or energy harvesting [2, 10, 11]. Compared to other stimuli, the application of an electrical stimulus offers a wide range of adjustable parameters including waveform, frequency, voltage, duty cycle, or electrode design, making it a stimulus of choice for controlling, programming, and monitoring the material's response. The actuation of EAPs is governed by the nature of the polymer [12, 13, 14]. The mechanical response of non‐ionic EAPs, including liquid crystals [15, 16, 17], dielectric elastomers [18, 19, 20], electrostrictive polymers [21, 22, 23], and gels [24, 25], is driven by electrostatic or Coulombic forces, often requiring high activation voltages (> 100 V) [12, 16, 26]. In contrast, ionic EAPs include conducting polymers [27, 28, 29, 30], ionic‐polymer metal composites [31, 32, 33], and ionic polymers and gels [34, 35]. These types of materials require much lower activation voltages (> 1 V), and their mechanical response is driven by diffusion/migration of ions and their hydration water inside and/or outside their polymeric network [14, 26, 36].

Of particular interest to this work are ionic EAPs, used for the formation of electro‐active hydrogels (EAHs). EAHs combine the aforementioned advantages offered through the use of an electrical stimulus with the advantages presented by hydrogels, such as high‐water content, conformability, and inherent biocompatibility [35, 37]. At the macro‐scale, Yang et al. demonstrated the fabrication of a bipedal millimeter‐sized walker made using poly(2‐acrylamido‐2‐methylpropanesulfonic acid‐co‐acrylamide) as the EAH, able to transport cargo of up to 25 times its weight [38]. Kwon et al. showed the possibility of using EAH cantilevers in microfluidic chips as valveless micro‐pumping systems [39]. Jackson et al. showcased the integration of an EAH cylinder into a catheter equipped with 3D interdigitated electrodes to create cardiovascular occlusions [40], which is necessary in numerous medical procedures, including acute hemorrhage, aneurysms, or cancer therapy [41, 42, 43, 44]. These examples, among others, demonstrated the versatility of applications for EAH and the impact that further development of these materials could bring [45, 46, 47, 48, 49]. However, most of the EAHs described to date in the literature, including the previous examples, displayed relatively simple 2D structures. The intrinsic and/or extrinsic structural complexity achieved hitherto has been limited by traditional manufacturing methods such as cutting, molding, or lithography. To lift these limitations, efforts have recently been made to fabricate EAHs using additive manufacturing (AM) methods, including direct ink writing (DIW) [50, 51, 52, 53, 54], and digital light processing (DLP) [55, 56, 57, 58]. 3D EAH structures have been demonstrated by Han et al. using DLP printing of poly(acrylic acid) as the EAH [56]. They showcased that various 3D designs of actuators could be printed using DLP, including a gripper, a brush‐like transporter, and a human‐like walker. The design of the humanoid shape allowed the millimeter‐sized figure to perform a walking motion at an average speed of 0.6 mm min^−1^ by bending its body, causing a shift in its center of gravity and a change in its anchoring point upon application of an electric field.

While the fabrication of complex 3D macro‐scaled actuators is now achieved through AM, those actuators displayed long response times, ranging from minutes to hours, which significantly hinders their practical applications [59, 60, 61, 62, 63].

The long actuation times typically seen for macro‐scaled hydrogel actuators come as a consequence of Fick's second law Equation (1)

(1) $$\frac{\partial c\left(x,t\right)}{\partial t}=D\frac{\partial^{2}c\left(x,t\right)}{\partial x^{2}}$$

where *c* is the concentration at a position *x* and time *t*, and *D* is the diffusion coefficient.

For a diffusion time *t_diff_* during which the diffusion takes place over a length scale *L*, Fick's second law can be approximated to:

(2) $$t_{diff}∼\frac{L^{2}}{D}$$

Thus, the kinetics of diffusion are typically proportional to the square of the linear dimension of the hydrogel [64, 65]. Consequently, one simple strategy to considerably reduce their response times entails a significant reduction of the EAHs’ dimensions to dramatically alter their surface‐to‐volume ratio.

Out of the available microfabrication methods, direct laser writing (DLW) by two‐photon polymerization (2PP) stands out, having been successfully demonstrated for the fabrication of complex micrometer‐sized hydrogel structures. In 2PP, a femtosecond laser is employed, and polymerization is confined within a small 3D volume (~100 × 100 × 300 nm), called a voxel. Fabrication by 2PP takes place in a voxel‐by‐voxel approach, endowing the structure with great extrinsic structural complexity, with feature sizes as small as ~300 nm [66, 67]. Additionally, 2PP enables facile control over a variety of laser power and design parameters to modulate the mechanical and chemical properties of the resulting microstructures, reaching unprecedented levels of intrinsic structural complexity. Using 2PP, a variety of hydrogels have been used to fabricate soft micro‐scaled actuators, displaying fast and programmable motions in response to external stimuli, such as changes in the hydration media or temperature, achieved through careful selection of fabrication parameters or multi‐material fabrication. Such examples include pillar arrays based on poly(ionic) liquids that achieved equilibrium swelling within a few seconds and showed fast temperature‐induced dehydration [68], hydrogel flowers that opened within 20 s in response to fructose addition (100 mM) [69], microcantilevers which bent within 0.133 s in response to a chemical cue (1 M NaCl) [70], or a transformer structure that converted from a humanoid‐like to a car‐like structure within 26 s in response to a pH change [71]. Although several of these hydrogel actuators employ ionizable monomers, including acrylic acid [71, 72, 73, 74, 75, 76, 77, 78, 79, 80, 81, 82, 83], 2‐acrylamido‐2‐methylpropane sulfonic acid [70], 2‐carboxyethyl acrylate [84], or 2‐(dimethylamino)ethyl methacrylate [85, 86], only recently has their ionizable nature been harnessed for electrically‐driven actuation [75]. As such, acrylic acid was used to create poly(acrylic acid‐co‐acrylamide) hydrogel microcilia arrays via 2PP. These microcilia were integrated onto a polyimide‐based microelectrode substrate, with four electrodes positioned around each array. It was demonstrated that these microcilia could bend within 0.7 s in response to an applied electric field of 10 000 V m^−1^ by individually addressing pairs of microelectrodes placed in the close proximity of the poly(acrylic acid) cylinders. As a result, bending enabled rotational motion with frequencies of up to 40 Hz, and was subsequently used to generate flow patterns within the system, with flow velocities as high as 250 µm s^−1^.

Herein, we develop novel poly(ionic) networks to create cationic and anionic EAHs (Figure 1). The mechanical response of EAHs is characterized as a function of hydrogel chemical composition, actuator design, and experimental parameters. Notably, we demonstrate for the first time the fabrication of gelatin methacrylate (GelMA)‐based EAH microstructures via 2PP, displaying a fast response (∼ 200 ms) to an electric field as low as 1 V mm^−1^. We demonstrate that the soft hydrogel nature of the EAH, displaying Young's moduli in the range of 0.4 to tens of MPa when hydrated, combined with optimal actuator geometry, is key for the realization of sustained, repeatable, and fast actuation (∼ 200 ms) under low electric fields (1 V mm^−1^). The photoresists used were based on well‐known pH‐responsive monomers, acrylic acid (AA, p*K_a_* (AA) ∼ 4.5) [87], and 2‐acrylamido‐2‐methyl‐1‐propanesulfonic acid (AMPS, p*K_a_* (AMPS) ∼ 1.9) [88], as well as (3‐acrylamidopropyl)trimethylammonium chloride (APTAC), a permanently positively charged monomer (Figure 1A). Gelatin methacrylate was used as a co‐macromer for AMPS and APTAC to enhance printability, water uptake, and electro‐actuation capabilities. Three photoresists were optimized, and successful fabrication via 2PP was assessed by means of geometric fidelity to the digital design. High‐fidelity microstructures with a feature size of approximately 1 µm were achieved. Micro‐cantilevers were then fabricated for electro‐driven actuation (Figure 1A–F), and their design optimized by systematically characterizing the influence of geometry (height, width, and length of the cantilever) on the degree of actuation, using the AA‐photoresist. The optimal cantilever geometry was determined to have a length of 80 µm, a width of 10 µm, and a height of 20 µm (Figure 1B), leading the p(AA) micro‐cantilevers to exhibit bending angles of up to 30.2 ±  3.1° (*n* = 384) in response to an electric field (6 V mm^−1^, 1 Hz). The optimal experimental conditions were studied for the three photoresists by examining the effect of electrolyte concentration, pH, electric field strength and direction on the degree of cantilever actuation. It was found that the optimal experimental conditions for the polyanionic micro‐cantilevers were 50 mM phosphate buffer solution of pH 7.4, with the p(AMPS) micro‐cantilevers displaying bending angles of 46.7 ± 4.8° (*n* = 1032) and response times of 100 ± 50 ms (*n* = 22) using an electric field of 5 V mm^−1^ (Figure 1E), resulting in angular actuation speeds of 467° s^−1^. In contrast, the optimal experimental conditions for the polycationic micro‐cantilevers were in a 50 mM KHP buffer solution of pH 3.2, with p(APTAC) micro‐cantilevers displaying bending angles of 11.1 ± 3.3° (*n* = 543) and response times of 200 ± 100 ms (*n* = 21), using an electric field of 4 V mm^−1^, resulting in angular actuation speeds of 55.5° s^−1^. To further expand this study, microfabrication of multi‐material electro‐actuators, combining poly(anionic) and poly(cationic) hydrogel segments, was achieved. These structures demonstrated selective electro‐actuation of individual structural elements within a single platform, governed by the material's chemistry and electrolyte pH, under a single electric field orientation. Finally, microstructures demonstrating actuation beyond single‐degree‐of‐freedom bending were explored using two different approaches. The first one used 2PP to spatially modulate the mechanical properties of vertical micro‐pillars, creating twisting motion of 22 ± 1° (*n* = 25) in response to the electric field. For the second approach, the electrical set‐up was modified to spatially modulate the electric field in 3D, enabling 360° steering of a micro‐pillar along a radial trajectory of 35 ± 5 µm (*n* = 20), as depicted in Figure 1G,H.

**FIGURE 1:**
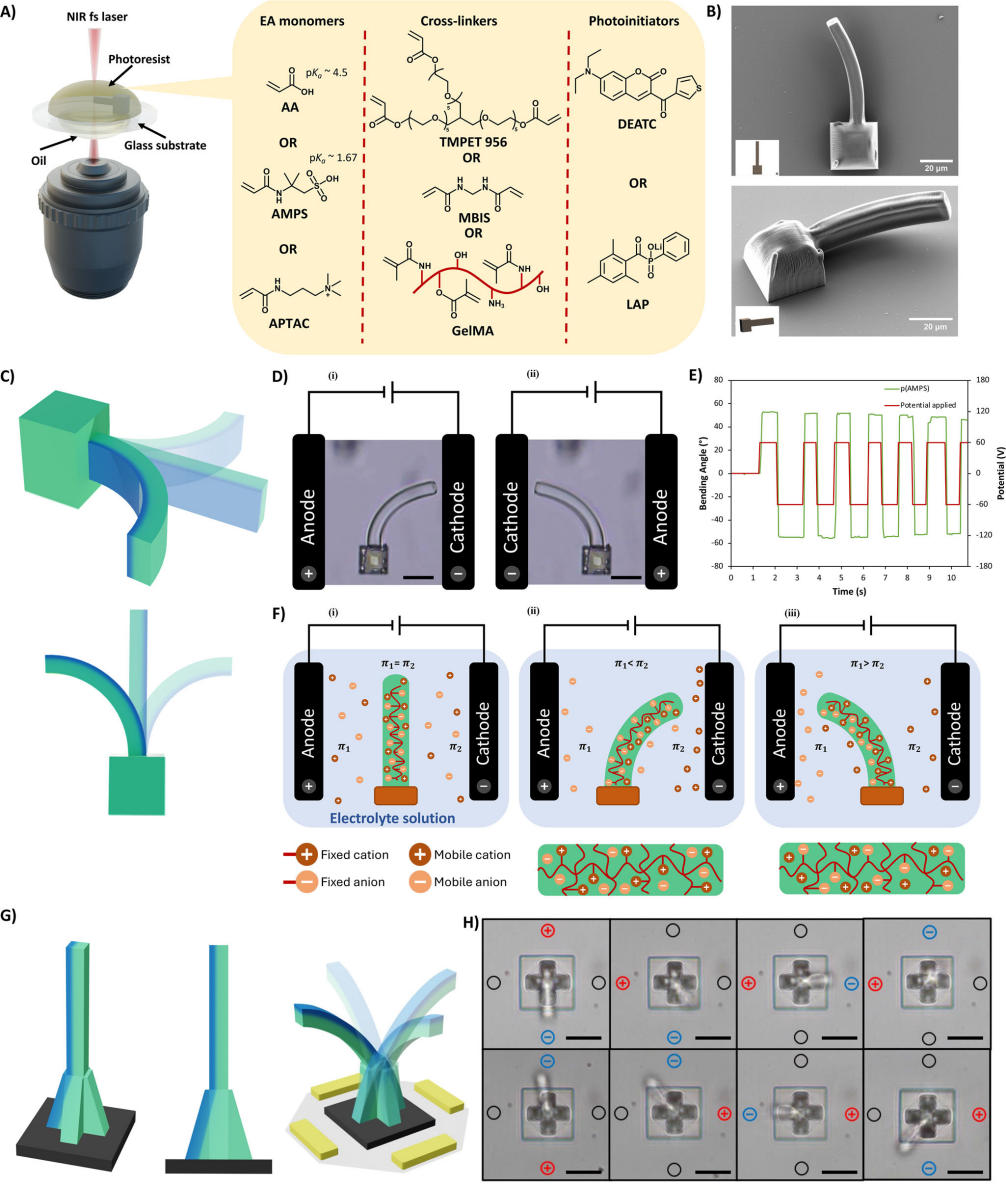
(A) Scheme of two‐photon polymerization (2PP) in oil immersion configuration for the fabrication of microstructures, and components of the photoresist: the electroactive (EA) monomers acrylic acid (AA), 2‐acrylamido‐2‐methylpropane sulfonic acid (AMPS), and (3‐acrylamidopropyl)trimethylammonium chloride (APTAC). The cross‐linkers trimethylolpropane ethoxylate triacrylate (TMPET 956), *N,N*‐methylenebis(acrylamide) (MBIS), and gelatin methacrylate (GelMA). The photoinitiators 7‐diethylamino‐3‐thenoylcoumarin (DEATC) and lithium phenyl‐2,4,6‐trimethylbenzoylphosphinate (LAP). The presence of a coverslip on top of the photoresist was omitted for clarity. (B) Scanning electron microscopy images of a microcantilever. (C) Schematic representation of cantilever actuation side‐ and top‐view. (D) Microscope images of microcantilevers fabricated by 2PP using the AMPS photoresist (Table 2), in 50 mM phosphate buffer with application of a potential (i) 60 V, and (ii)−60 V. (E) Bending angle of a micro‐cantilever (80 × 10 × 20 µm^3^) fabricated using the AMPS photoresist in 50 mM phosphate buffer with application of an electric field (6 V mm^−1^). (F) Schematic representation of the mechanism of electro‐actuation (i) before and after applying an electric stimulus to the electrolyte solution in the case of an anionic (ii) and cationic (iii) EAH, leading to redistribution of charges in and out of the hydrogel. (G) Schematic representation and (H) sequential microscopy images of a vertical square pillar (40 × 40 × 120 µm^3^) having a cross‐shaped base structure (40 × 40 × 40 µm^3^), depicting 360^○^ anticlockwise circular motion upon programmable actuation using a four‐electrode system. Scale bars represent (D) and (H) 30 µm.

## 2 Results and Discussion

### 2.1 Fabrication of Microstructures via 2PP

The response speed of electrically driven hydrogel actuators is largely governed by ion diffusion through the hydrogel network. To improve actuation speed, reducing the ion diffusion path and increasing the ion diffusion rate are effective strategies. Prior work in stimuli‐responsive hydrogels has taken advantage of creating highly porous networks to speed up actuation [89]. A different strategy is employed here, where the hydrogel actuator's size is decreased to micrometer dimensions by employing direct laser writing microfabrication. This significantly boosts actuation speed by shortening the ion diffusion path within the hydrogels Equation (2).

To ensure a high density of ionized/ionizable groups within the hydrogel network, photoresist formulations for DLW were developed incorporating a high content (> 40 wt.%) of the ionized/ionizable monomer (Tables 1, 2, 3). As such, formulations based on acrylic acid (AA) and 2‐acrylamido‐2‐methylpropane sulfonic acid (AMPS) were optimized for the realization of anionic polyelectrolyte networks, while (3‐acrylamidopropyl)trimethylammonium chloride (APTAC) was used for the polycationic EAH (Figure 1A). Anionic monomers will become negatively charged, and therefore electro‐active, at pH values above their p*K_a_*. Photoresist formulations based on acrylic acid (AA, p*K_a_* (AA) ∼ 4.5), a well‐known pH‐responsive monomer, have been previously demonstrated for DLW‐fabrication of micro‐actuators [71, 72, 73, 76, 77, 78, 79, 80, 81, 82, 83].

**TABLE 1:** Composition of AA‐based photoresist used for 2PP.

Compound	Molecular weight [g/mol]	Moles [mmol]	Mass [mg]	Ratio [mol.%]
Acrylic acid	72.06	1.39	100.0	69.8
Ethoxylated trimethylolpropane triacrylate	956.00	0.59	559.9	29.4
7‐Diethylamino‐3‐thenoylcoumarin	327.40	0.016	5.1	0.8

**TABLE 2:** Composition of AMPS‐based photoresist used for 2PP.

Compound	Molecular weight [g/mol]	Moles [mmol]	Mass [mg]	Ratio [wt%]
2‐acrylamido‐2‐methyl‐1‐propanesulfonic acid	207.25	1.22	253.0	42.9
*N,N*‐methylenebis(acrylamide)	154.17	0.12	18.8	3.2
Gelatin methacrylate	—	—	60.0	10.2
Lithium phenyl‐2,4,6‐trimethylbenzoylphosphinate	294.20	0.03	9.0	1.5
Dimethyl sulfoxide	78.13	2.11	165.0	28.0
Deionized water	18.02	4.67	84.2	14.2

**TABLE 3:** Composition of APTAC‐based photoresist used for 2PP.

Compound	Molecular weight [g/mol]	Moles [mmol]	Mass [mg]	Ratio [wt%]
(3‐acrylamidopropyl)trimethylammonium chloride	206.70	1.63	337.0	57.1
*N,N*‐methylenebis(acrylamide)	154.17	0.12	18.8	3.2
Gelatin methacrylate	—	—	60.0	10.2
Lithium phenyl‐2,4,6‐trimethylbenzoylphosphinate	294.20	0.03	9.0	1.5
Dimethyl sulfoxide	78.13	2.11	165.0	28.0

Compared to AA, 2‐acrylamido‐2‐methylpropane sulfonic acid (AMPS, p*K_a_* (AMPS) ∼ 1.67), presents a more versatile electro‐active monomer as it is ionizable across a broader range of pH values due to its low p*K_a_*. (3‐acrylamidopropyl)trimethylammonium chloride is a permanently charged cationic monomer enabling the realization of a poly(cationic) electro‐actuator. The crosslinkers used in the photoresist formulations include ethoxylated trimethylolpropane triacrylate M_n_ = 956 g mol^−1^ (TMPET 956), *N,N*‐methylenebis(acrylamide) (MBIS), and gelatin methacrylate (GelMA) (Figure 1A). GelMA's role in the composition is threefold; a crosslinker due to multiple polymerizable groups in the chain, a functional macromer having an estimated isoelectric point around pH 7‐9 [90], and an optimal host polymer network, allowing for tunable hydrophilicity and mechanical properties.

Micro‐fabrication using the selected anionic and cationic EAH photoresist formulations (Tables 1, 2, 3) was explored by screening 2PP fabrication parameters (laser power, scanning speed, hatching, and slicing) to produce simple 3D microstructures (e.g., cuboids). Initially, fabrication of 10 × 10 × 4 µm^3^ cuboids was tested over a range of different laser powers, scanning speeds, and slicing and hatching distances (Figures 2A–C and S1). Successful micro‐fabrication for hatching and slicing distances of 0.2 µm was achieved using laser powers ranging from 22.5 to 35 mW at 10 mm s^−1^ for the AA‐based photoresist, 32.5 to 50 mW at 1 mm s^−1^ for the AMPS‐based photoresist, and 20 to 47.5 mW at 2.5 mm s^−1^ for the APTAC‐based photoresist (Figure 2A–C). Below these ranges, either no polymerization occurred, or the resulting cuboids presented a poor printing fidelity, exhibiting rounded corners and considerably smaller sizes compared to the design (Figure 2A–C). Above these ranges, overheating of the photoresist resulted in micro‐explosions, which compromised the resolution and printing fidelity of the structures.

**FIGURE 2:**
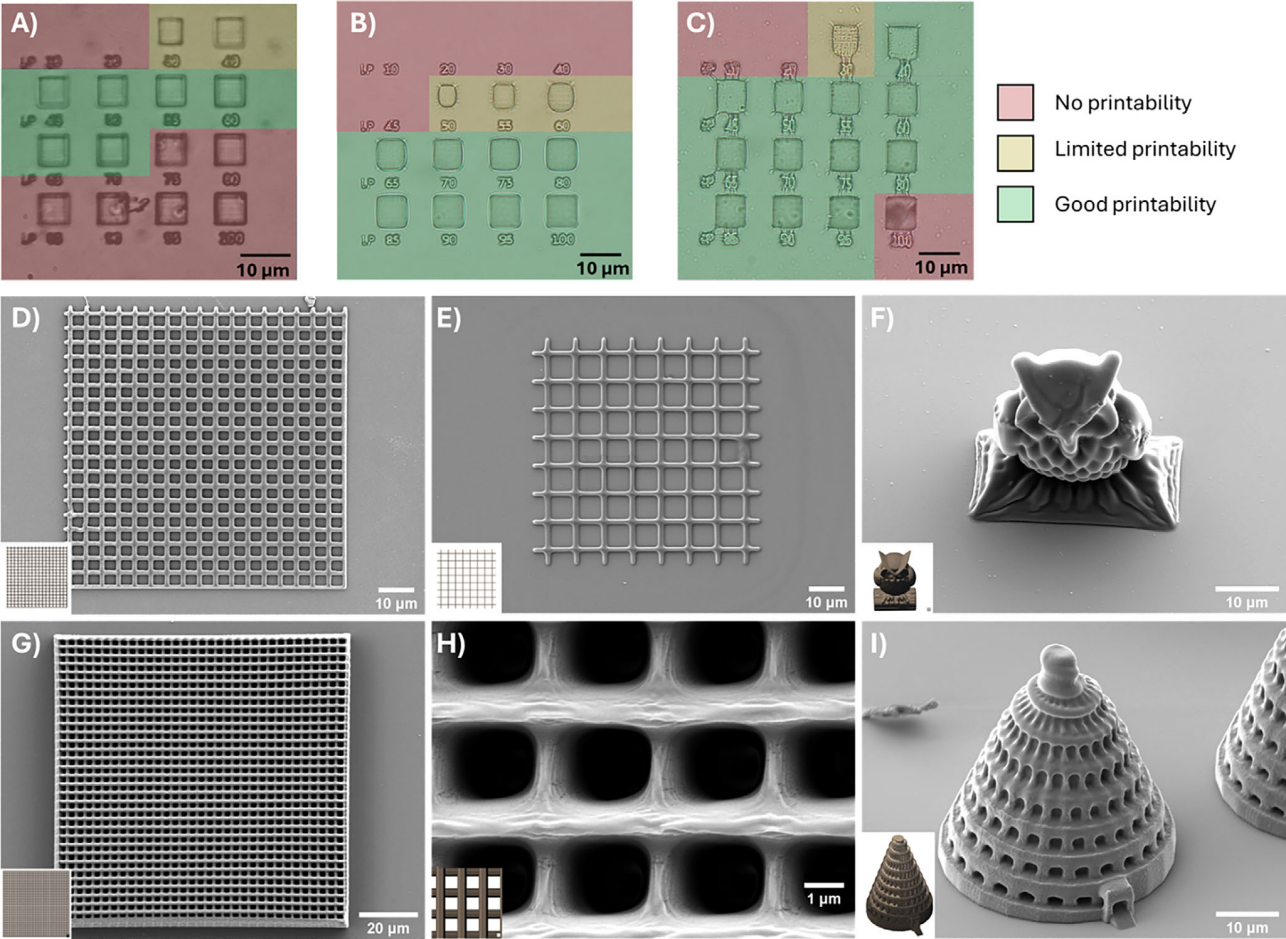
Microscope images of cuboids (10 × 10 × 4 µm^3^) fabricated with varying laser power and slicing distance of 0.2 µm using the: (A) AA‐, (B) AMPS‐, and (C) APTAC‐based photoresists (Tables 1, 2, 3). Scanning electron microscopy images of: grids fabricated using (D) the AMPS photoresist and (E) the APTAC photoresist; (F) an owl fabricated using the APTAC photoresist (30 mW and 10 mm s^−1^); (G) a 16‐layer woodpile, (H) a zoom of the woodpile, and (I) a Babylon Tower, fabricated using the AA photoresist (22.5 mW and 10 mm s^−1^). The scale bars represent 10 µm in (A–F) and (I), 20 µm in (G), and 1 µm in (H).

To showcase the precision and structural complexity achieved with 2PP, a variety of 3D structures were fabricated (Figures 2 and S2–S4). Grid structures covering an area of 160 µm^2^ were successfully fabricated at 50 mW and 1 mm s^−1^ using the AMPS photoresist, demonstrating minimum feature sizes of 1.35 ± 0.05 µm (Figure 2D). Grid structures (100 × 100 µm^2^, made from lines of 1 µm designed width) were fabricated at 30 mW and 10 mm s^−1^, yielding feature sizes down to 1.23 ± 0.09 µm using the APTAC photoresist (Figure 2E). Successful fabrication of a woodpile structure of up to 16 layers, made of 1 µm diameter cylinders with a 2 µm periodicity, was demonstrated with fidelity to the initial design and 0.80 ± 0.16 µm features, using fabrication parameters of 30 mW and 10 mm s^−1^ with the AA photoresist (Figure 2G,H). Due to the inherent softness of the materials produced from the AMPS‐ and APTAC‐based photoresists, the resulting structures exhibited enlarged feature sizes compared to AA‐based structures. More complex structures were also successfully fabricated using these three photoresist formulations (Figures 2 and S2–S4), demonstrating the higher resolution and degree of structural complexity achieved with 2PP, compared to other types of photo‐induced 3D printing such as digital light processing (highest achievable resolution reached ∼ 5 µm) [55].

Arrays of 10 × 10 × 4 µm^3^ cuboids were also fabricated by 2PP for each photoresist to chemically and mechanically characterize the resulting microstructures. All arrays were fabricated at a slicing and hatching distance of 0.2 µm. The AA‐based array was fabricated at 10 mm s^−1^ and with laser powers ranging from 20 to 40 mW in 2.5 mW increments (Figure 3A,B). The AMPS‐ and APTAC‐based arrays were fabricated at 10 mm s^−1^ and with laser powers ranging from 15 to 35 mW in 2.5 mW increments. First, the chemical composition of the micro‐cubes was assessed by measuring their Raman spectra, together with the spectra of the corresponding unpolymerized photoresists (Figures 3C and S5–S7). This allowed for the estimation of the monomer/macromer to polymer conversion (i.e., degree of conversion) by assessing the conversion of the C═C bond (Figure 3D). In the case of the AA‐based microcubes, a clear decrease in the intensity of the C═C band centered at 1606 cm^−1^ was observed compared to the unpolymerized AA‐photoresist, while the intensity of the C═O band remained constant at 1733 cm^−1^. For the GelMA‐containing compositions, the C═O band appeared at 1655 cm^−^^1^ for the AMPS photoresist and at 1661 cm^−^^1^ for the APTAC photoresist, and its position in the spectrum remained similar upon 2PP. The C═C band was observed at 1633 cm^−^^1^ for AMPS and 1629 cm^−^^1^ for the APTAC photoresist, respectively (Figure 3C). The ratio between the intensity of the C═O and C═C bands was used in the determination of the degree of conversion, however, the proximity of the two bands in the case of p(AMPS) and p(APTAC) microstructures could introduce errors in this calculation. The degree of conversion was calculated in the range of 54% to 63% for the AA‐based microstructures, 68% to 70% for the AMPS microstructures, and 80% to 85% for the APTAC microstructures, respectively (Figure 3D). The increase in the laser power from 20 to 37.5 mW increased the degree of conversion in the case of AA‐structures (from 54% to 63%), but had little effect on the degree of conversion calculated for the AMPS and APTAC‐based structures. The Raman spectra of both the AMPS photoresist and its corresponding structures displayed bands corresponding to the stretching vibration of ─SO_3_ at 1045 and 1053 cm^−1^, ─SO at 1122 and 1109 cm^−1^, respectively, demonstrating the presence of the sulfonic acid moiety within the 2PP fabricated structures (Figure S6) [91, 92]. In the case of the APTAC photoresist and its corresponding microstructures, characteristic bands of the quaternary amine were present in their Raman spectra, such as symmetric stretching vibration of ─(CH_3_)N^+^ at 760 cm^−1^, asymmetric stretching vibration of ─(CH_3_)N^+^ at 960 cm^−1^, –C–N asymmetric stretching vibration at 1250 cm^−1^ (Figure S7) [92, 93].

**FIGURE 3:**
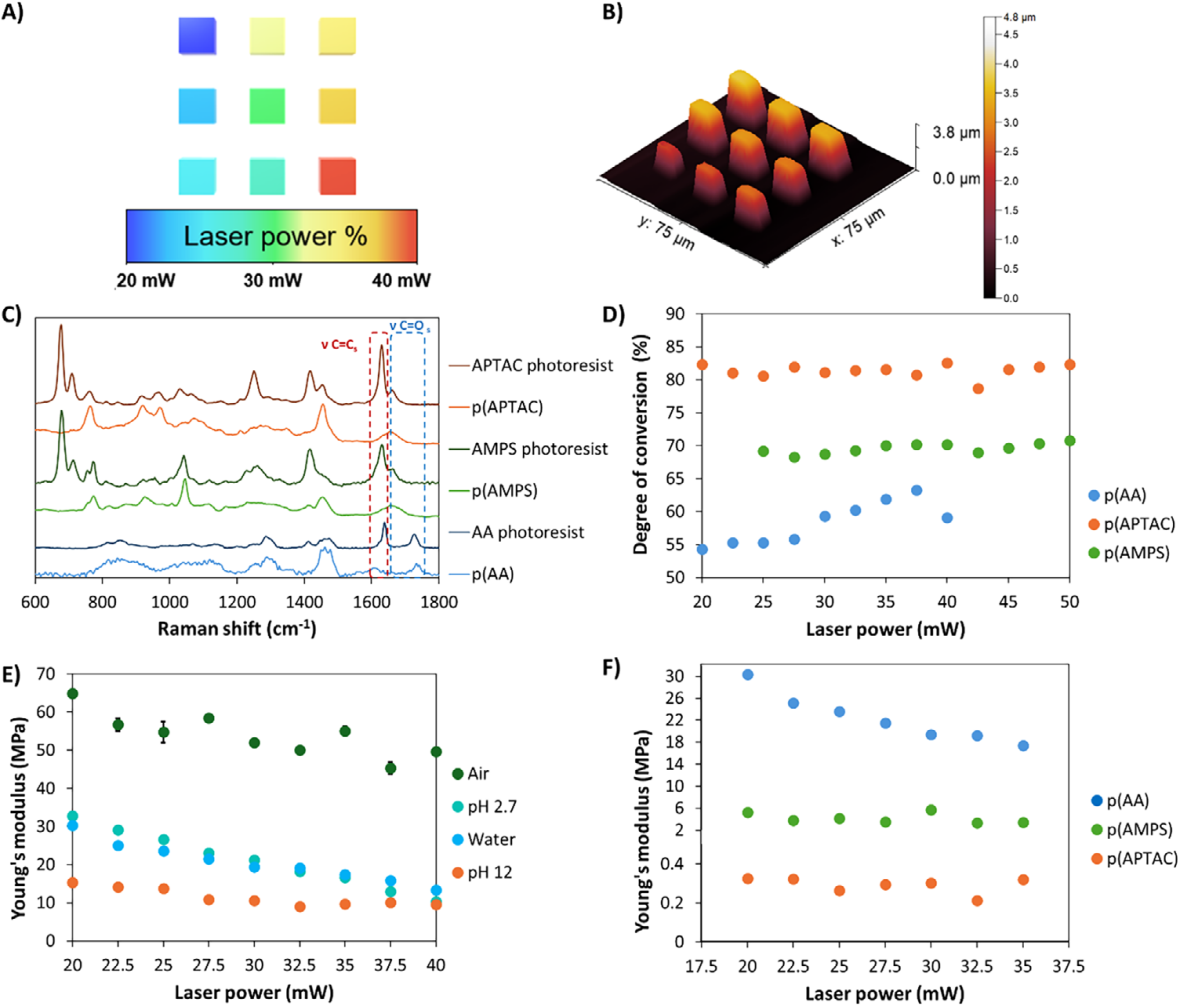
(A) Map depicting the p(AA) micro‐cube array fabricated using increasing laser powers, from 20 to 40 mW, in 2.5 mW increments. (B) Topography of the p(AA) micro‐cuboids in their dry state obtained using AFM. (C) Raman spectroscopy of the photoresists and of the corresponding microcuboids fabricated at 35 mW. (D) Effect of laser power on the degree of conversion for microstructures fabricated using the AA, AMPS, and APTAC photoresists. (E) Effect of laser power on the Young's modulus of p(AA) micro‐cuboids in the dry state, when hydrated at pH 2.7 (HCl solution), deionized water, and at pH 12 (NaOH solution), respectively. (F) Effect of laser power on the Young's modulus for p(AA), p(AMPS), and p(APTAC) micro‐cuboids in deionized water.

Next, the swelling and mechanical properties of the microstructures (10 × 10 × 4 µm^3^ cuboids) were investigated using atomic force microscopy (AFM), through height and Young's modulus measurements of the microstructures upon hydration in deionized water, pH 2.7 and pH 12 solutions (Figures 3E,F and S8–S13). In all cases, the height of the micro‐cuboids increased with increasing laser power used during 2PP fabrication. The p(AA) microstructures reached a maximum height at 35 mW and slightly decreased beyond this point. Increasing the laser power increases the effective voxel size and the crosslinking density of the polymer network. For example, the height of the p(AA) cuboids fabricated at 20 and 35 mW in their dry state was determined to be 2.28 ± 0.01 µm (*n* = 3) and 3.73 ± 0.01 µm (*n* = 3), respectively, which correspond to ∼ 57% and ∼ 93%, respectively, of the designed height of 4 µm (Figure S11). Upon hydration in an aqueous solution of pH 2.7 (below the p*K_a_* of AA), the height of the cubes increased to reach 2.99 ± 0.02 µm (*n* = 3) and 4.33 ± 0.01 µm (*n* = 3), for cubes fabricated at 20 and 35 mW, respectively, which correspond to an increase of 30.9% and 16% from their dry state (Figure S11). These results are consistent with lower laser power producing a polymer network of lower crosslinking density, which has higher swelling abilities, and higher laser power producing cuboids of higher crosslinking density, which have lower swelling abilities. A similar trend was observed upon hydration in a pH 12 solution (at pH above the p*K_a_* of AA) where the height of the cuboid structures reached 3.54 ± 0.05 µm (*n* = 3) and 4.69 ± 0.04 µm (*n* = 3) for cuboids fabricated at 20 and 35 mW, respectively, corresponding to an increase of 54.8% and 25.7% from their dry state (Figure S11). This greater height increase compared to the pH 2.7 solution is owed to the ionization of the polymeric network in the basic environment. The ionization of the AA units significantly enhances the polymer hydrophilicity and generates electrostatic repulsion between the charged polymeric chains. These results are consistent with the variation of Young's modulus of the p(AA) microstructures as a function of the pH of the hydration media (Figure 3F). The Young's modulus of the p(AA) microstructures reached a maximum of 30.3 ± 0.4 and 32.8 ± 0.6 MPa when hydrated in deionized (DI) water and in a pH 2.7 solution, respectively. The Young's modulus significantly decreased at pH 12, with the highest Young's modulus being 15.3 ± 0.3 MPa. As previously mentioned, ionization of the AA units enhances the swelling capabilities of the p(AA) microcubes, which in turn reduces the stiffness of the structures. The effects of pH on the physical properties of the p(AMPS) and p(APTAC) microcuboids (10 × 10 × 4 µm^3^, slicing of 0.2 µm) were also studied through AFM measurements (Figures S12 and S13). Upon hydration in solution at pH 12, the p(AMPS) microstructures swelled up to 38% compared to their hydrated state in DI water. On the contrary, the height of the micro‐cubes decreased slightly at pH 2.7, with a maximum shrinking of 6.0% relative to their hydrated state in DI water. The p(APTAC) structures displayed a relatively constant height in both DI water and pH 2.7 solution, while shrinking of up to 18% was observed when the structures were hydrated in a pH 12 solution. Young's modulus measurements demonstrated that the p(AMPS) structures were stiffer in pH 2.7 conditions, reaching a modulus of 10.6 ± 0.3 MPa, compared to 6.39 ± 0.09 and 5.74 ± 0.05 MPa in pH 12 and in DI water, respectively (Figure S12). The Young's modulus of the p(APTAC) structures reached a maximum of 387.7 ± 5.0 kPa at pH 12, and displayed similar values when the p(APTAC) microstructures were hydrated in DI water, with a maximum of 326.3 ± 2.7 kPa. However, when exposed to a solution of pH 2.7, the p(APTAC) microstructures displayed a significantly lower Young's modulus, reaching 150.3 ± 0.6 kPa (Figure S13). In the case of the p(AMPS) and p(APTAC) microstructures, the changes in pH can also affect the overall charges of the GelMA component, having its isoelectric point estimated ∼ pH 7–9 [90], meaning that in pH 12 conditions, above its isoelectric point, GelMA's net charge is negative. Therefore, at pH 12, while the height of the p(AMPS) microstructures is increasing compared to pH 2.7 and DI water conditions, the height of the p(APTAC) is decreasing. This can be rationalized by an increase in negative charges within the p(AMPS) microstructures at pH 12, yielding higher charge repulsions and therefore swelling compared to pH 2.7 and DI water conditions. In contrast, higher ionic interactions between the negatively charged GelMA and the positively charged p(APTAC) at pH 12 cause a contraction of the polymer network and a decrease in the height of the microstructures. The Young's modulus of p(AMPS) and p(APTAC) also displayed opposite trends as a function of pH, with exposure to pH 2.7 conditions showing a stiffening of the p(AMPS) structures and a softening of the p(APTAC) structures, which is similarly rationalized by the changes of the overall charge of GelMA, which dictate the ionic interactions within the microstructures and therefore the swelling of the network and its mechanical properties.

### 2.2 Electro‐Actuation of Micro‐Cantilevers

To investigate the electro‐actuation capabilities of the microstructures, micro‐cantilevers were fabricated via 2PP. These were composed of a base (30 × 30 × 40 µm^3^) to which a cantilever (80 × 20 × 10 µm^3^) was suspended (Figures 1B,C, and S14). The base was fabricated at 50 mW and 10 mm s^−1^, while the fabrication parameters of the cantilever were optimized at a slicing distance of 0.3 µm and a scanning speed of 10 mm s^−1^. The laser power for fabrication was investigated in the range of 15–50 mW, with 30 mW being selected for all p(AA) micro‐cantilever fabrication, as it was the lowest power at which the structures could withstand post‐fabrication development while being able to be self‐supporting upon hydration.

The electro‐actuation of polyelectrolyte hydrogels in electrolyte solutions where the ion concentration is lower than that of the ionized groups in the hydrogel (i.e., cases of low ionic strength) is commonly explained by the dynamic enrichment and depletion mechanism [45, 59, 60]. In the case of a polyanionic hydrogel strip, upon hydration in an electrolyte solution, mobile ions are absorbed into its charged polymeric network to neutralize polymer‐bound charges (Figure 4Ai). Upon applying an electric field, mobile ions redistribute both inside and outside the hydrogel (Figure 4Aii), resulting in an ion enrichment at the hydrogel side facing the cathode (−) (Figure 4Aiii) and an ion depletion at the hydrogel side facing the anode (+) (Figure 4Aiii). This redistribution of ions creates an osmotic pressure difference between the two interfaces of the hydrogel, prompting water to diffuse through the polymeric network to restore equilibrium (Figure 4Aiii) [45, 61, 62, 63]. This difference in water intake induces the bending of the hydrogel toward the cathode (−) (Figure 4Aiv). According to this mechanism, the bending direction of an EAH is determined by the charge of its polymeric network: a polyanionic hydrogel will bend toward the cathode (−), while a polycationic hydrogel will bend toward the anode (+), aligning with the electrode that matches the charge of the polymer network (Figure 4B).

**FIGURE 4:**
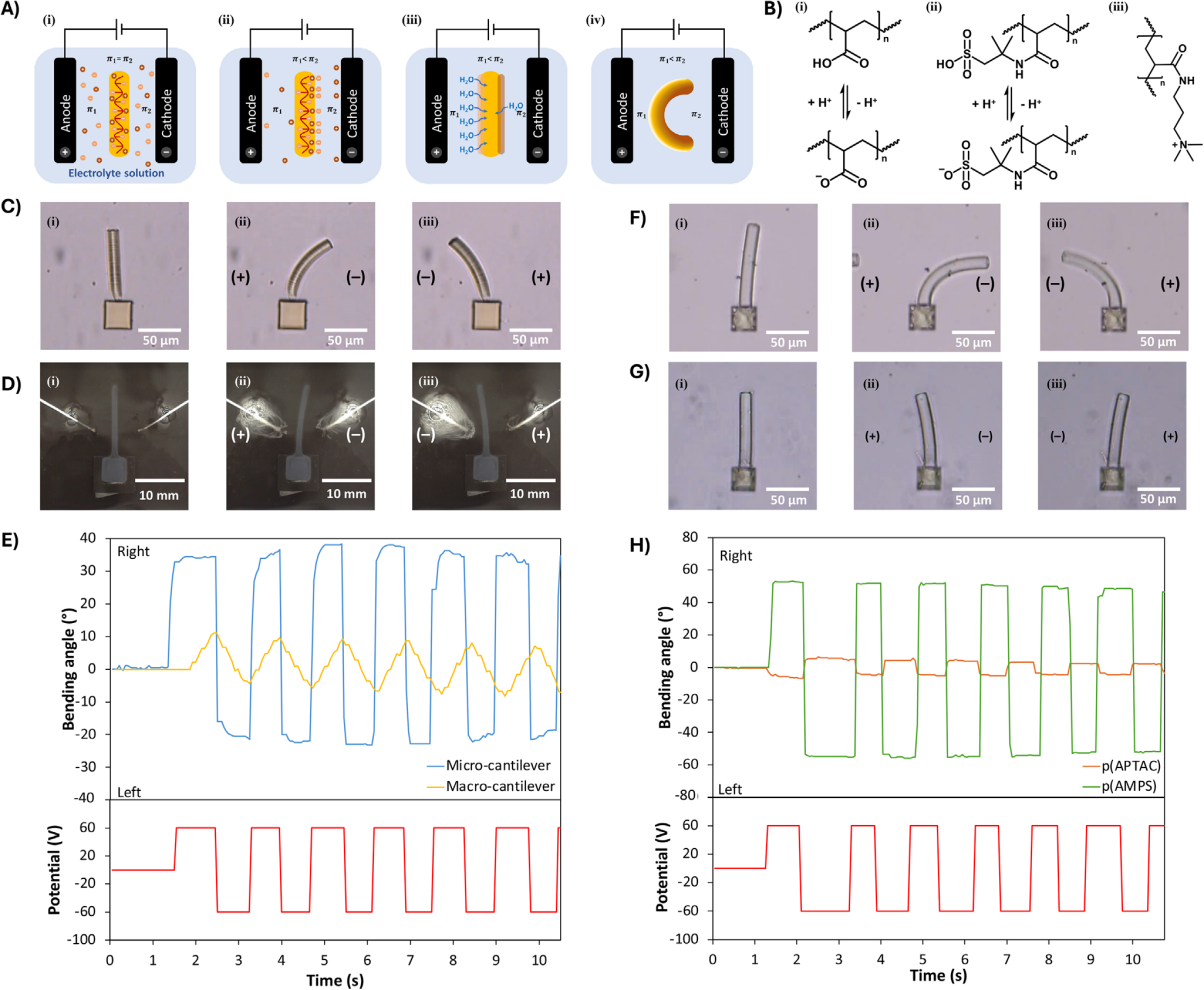
(A) Schematic representation of the bending of a polyanionic hydrogel strip driven by the dynamic enrichment and depletion mechanism. Adapted from Jin et al. [95]. (B) Chemical structure of (i) p(AA), (ii) p(AMPS) showing ionization of the polymeric networks upon pH changes, and (iii) p(APTAC) with its permanently charged polymeric network. (C) and (D) Microscopy images of the p(AA) micro‐cantilever and macro‐cantilever, respectively, (i) before applying an electric field, (ii) after applying an electric field (6 V mm^−1^, 1 Hz), and (iii) upon reversing the polarity of the electrodes. (E) Comparison of the bending angle of a micro‐cantilever (80 × 10 × 20 µm^3^) and macro‐cantilever (8 × 1 × 2 mm^3^) over six polarity cycles. (F) and (G) Microscopy images of the p(AMPS) and p(APTAC) micro‐cantilever, respectively, (i) before applying an electric field, (ii) after applying an electric field (6 V mm^−1^, 1 Hz), and (iii) upon reversing the polarity of the electrodes. (H) Comparison of the bending angle of the p(AMPS) and p(APTAC) micro‐cantilevers over six polarity cycles.

First, the p(AA)‐based micro‐cantilevers were tested for their electro‐actuation capabilities. The micro‐cantilevers were immersed in a 50 mM phosphate buffer of pH 7.4, and two Pt electrodes were placed on either side of the cantilevers at a constant distance, d_A‐C_, of 10 mm, so that the electric field lines were perpendicular to the micro‐cantilevers (Experimental set‐up and Figure S16C) [53, 94]. The micro‐cantilevers were then subjected to an electric field of 6 V mm^−1^, with the voltage polarity changed every 1 s (1 Hz) using a programmable switch. The mechanical response of the micro‐cantilevers to the electric field was recorded over time in top view using optical microscopy, and assessed by measuring their bending angle from their initial position (Movie S1, Bending angle measurements and Figure S16D). Before applying the electric field, the micro‐cantilevers remained straight, with a bending angle of zero (Figure 4Ci). Upon application of the electric field (6 V mm^−1^), the micro‐cantilevers exhibited a fast and pronounced mechanical response toward the cathode (−), reaching a bending angle of 32.1 ± 2.7° (*n* = 201) (Figure 4Cii). When the polarity of the electrodes was switched, the micro‐cantilever reversed its direction of bending with a similar bending amplitude (−28.2 ± 2.0° (*n* = 183)) toward the right, corresponding to the new position of the cathode (−) (Figure 4Ciii). The electro‐actuation of the p(AA) was attributed to its negatively charged polymeric network, given that the pH of the electrolyte solution was above the p*K_a_* of p(AA) of 4.5 (Figure 4B) [87]. This result is outstanding in terms of hydrogel electro‐actuator performance, whereas previously reported structures typically required equilibrium actuation times of minutes to hours [34, 38, 45, 56, 58]. By moving from typical millimeter‐sized to micrometer‐sized electro‐actuators as reported in this work, the ion diffusion path was dramatically reduced, thus increasing the ion/hydration water diffusion speed. While just recently the electro‐actuation of micro‐sized p(AA) pillars was demonstrated by Sitti and co‐workers, the present system operates on timescales consistent with their demonstrated EAH actuation, while requiring electric fields that consistently lower (1 V mm^−1^ versus ∼10 V mm^−1^) [75].

To directly compare the performances of our micro‐scale electro‐actuators fabricated via DLW by 2PP with previously reported macro‐scale electro‐actuators, a mm‐sized cantilever (8 × 1 × 2 mm^3^), respecting the same aspect ratio as the AA‐based micro‐cantilever (80 × 10 × 20 µm^3^), was fabricated via digital light processing (DLP) (Table S1) using an AA‐based photoresist compatible with this additive manufacturing method, as previously reported (Table 4) [55, 56]. In this context, the crosslinker concentration was reduced from 29.4 mol% (for 2PP) to 1.1 mol% (for DLP), which should further enhance the hydration capabilities of the AA hydrogel. Such small crosslinker concentrations are not suitable for DLW via 2PP, where micro‐fabrication occurs in a voxel‐by‐voxel manner; hence, each volume element needs to contain a sufficiently high crosslinker concentration to result in successful fabrication of a solid structure. Following a similar experimental procedure and conditions (50 mM phosphate buffer solution of pH 7.4, and electric field of 6 V mm^−1^, 1 Hz), the electro‐actuation capabilities of the macro‐scale cantilever were assessed and displayed a similar behavior to the p(AA) micro‐cantilever (Figure 4D, Movie S2). The kinetics and amplitude of actuation were considerably different for the macro‐scale compared to the micro‐scale cantilevers (Figure 4E). As such, the micro‐sized cantilever reached its plateau, equilibrium bending angle of 28.9 ± 7.1° (*n* = 128) within 150 ± 50 ms (*n* = 18) and remained stable for the entire duration of the applied electric field (1 s). Whereas the millimeter‐sized cantilever did not reach a plateau within the timeframe before the polarity was reversed (1 s) and showed a lower bending angle of 7.9 ± 2.0° (*n* = 10). Instead, it required 34.2 ± 3.0 s (*n* = 8) to reach a bending angle of 29°, similar to that of the micro‐sized cantilever and reached its plateau value of 30.7 ± 1.5° (*n* = 1317) within 41.3 ± 3.3 s (*n* = 8) upon applying an electric field of 6 V mm^−1^ at 16.7 mHz (Movie S2, Figure S17). Thus, by reducing the dimensions of the micro‐cantilever by a factor of 10^2^, the actuation time was effectively reduced by a factor of 228. This represents a greater improvement than the recently reported EAH microstructures, which exhibited approximately a 100‐fold reduction in their actuation times relative to their macroscale counterparts [75]. It is worth noting that the camera used to record the electro‐actuation of the micro‐cantilevers allows for a capture frame rate of up to 20 frames per second, resulting in a time interval of 50 ms between subsequent frames. Therefore, due to the rapid actuation times and the camera's frame rate, the response times of the micro‐cantilevers are reported in discrete intervals of 50 ms. While these measurements clearly demonstrate the fast actuation of the microstructures, the use of a high‐speed camera in future studies would enable a more precise determination of the response times.

**TABLE 4:** Composition of AA‐based photoresist used for DLP.

Compound	Molecular weight [g/mol]	Moles [mmol]	Mass [mg]	Ratio [mol%]
Acrylic acid	72.06	62.49	4503.2	98.6
Poly(ethylene glycol) diacrylate	700.00	0.69	481.2	1.1
Phenylbis(2,4,6‐trimethylbenzoyl)phosphine oxide	418.50	0.20	83.7	0.3

Similarly, micro‐cantilevers (80 × 10 × 20 µm^3^) based on different monomers (AMPS and APTAC) were fabricated using DLW by 2PP. Due to the excessive swelling of the materials produced from the AMPS‐ and APTAC‐based photoresists, resulting in the delamination of the structures, the p(AMPS) and p(APTAC) micro‐cantilevers were fabricated in a two‐step process. The base of the micro‐cantilevers was fabricated using IP‐L, a commercially available resin, at 30 mW and 10 mm s^−1^. Similar to the p(AA) micro‐cantilevers, the fabrication of the suspended cantilever was achieved through optimization of the fabrication parameters. A slicing distance of 0.3 µm, a laser power range of 22.5–32.5 mW, and a scanning speed of 1.0 mm s^−1^ were compatible with the p(AMPS) fabrication, while a laser power range of 25–45 mW and a scanning speed of 2.5 mm s^−1^ were deemed successful for p(APTAC) cantilever fabrication. All p(AMPS) cantilevers subsequently investigated were fabricated at a set scanning speed of 1.0 mm s^−1^ and a laser power of 27.5 mW, whereas a scanning speed of 2.5 mm s^−1^ and a laser power of 37.5 mW were selected for the fabrication of p(APTAC) micro‐cantilevers.

The p(AMPS) and p(APTAC) micro‐cantilevers were then tested for their electro‐actuation capabilities under the same experimental conditions (Figure 4F–H, Movie S1). Upon application of the electric field (6 V mm^−1^, 1 Hz), the p(AMPS) micro‐cantilever exhibited a fast (100 ± 50 ms, *n* = 22) and pronounced mechanical response, bending toward the cathode (−), with a bending angle of 43.5 ± 8.0° (*n* = 498) and a bending angle of −46.3 ± 7.3° (*n* = 454) when the polarity was reversed (Figure 4Fii, iii), corresponding to an overall average bending angle of 44.9 ± 7.8°. The electro‐actuation of the p(AMPS) was attributed to its negatively charged polymeric network, given that the pH of the electrolyte solution was above the p*K_a_* of p(AMPS) of 1.67 (Figure 4Bii). The p(APTAC) micro‐cantilever also demonstrated electro‐actuation capabilities with a fast (200 ± 100 ms, *n* = 21) mechanical response toward the anode (+), with bending angles of 5.0 ± 2.3° (*n* = 309) (Figure 4Gii) and a bending angle of −5.4 ± 1.6° (*n* = 286) when the polarity was switched (Figure 4Giii). The electro‐actuation of the p(APTAC) was attributed to its positively and permanently charged polymeric network (Figure 4Biii).

In all cases, the p(AA), p(AMPS), and p(APTAC) micro‐cantilevers reached their maximum bending within 200 ms upon the application of the electric field (6 V mm^−1^). Such a rapid response excludes an electro‐actuation mechanism driven by ionization differences within the polymeric network, which would be caused by an electrochemically generated pH gradient within the electrolyte solution between the electrodes. The kinetics of such a mechanism would depend on the diffusion of hydronium and hydroxide ions within the electrolyte solution. In such a case, given the available ion mobility values (Table S9), the estimated timescales are ∼ 2.3–4.0 s for ion migration from the electrode to the nearest micro‐cantilever interface (Table S10), which does not match the observed response times. In contrast, if the electro‐actuation of the micro‐cantilevers is assumed to originate from an electrically generated osmotic pressure difference between the two hydrogel interfaces, the counterion migration times between these interfaces are estimated to be in the range of ∼ 4.6–32.1 ms (Table S11), which is closer to the observed response times.

### 2.3 Effect of Geometry Design on Micro‐Cantilever Actuation

To further understand and optimize the electro‐actuation of the structures fabricated using 2PP, the effects of geometry design and experimental parameters were studied. The influence of the length, height, and width of the micro‐cantilever's beam on its resulting electro‐actuation was systematically characterized for the p(AA) micro‐cantilevers. The different micro‐cantilevers were fabricated using the same parameters and fabrication protocol described in the previous section, and their electro‐actuation was assessed under the following experimental conditions: 50 mM phosphate buffer solution of pH 7.4, and 4 V mm^−1^ at 1 Hz. Therefore, the results of this study are exclusively based on mechanical considerations and can be generalized to other micro‐cantilever beams, irrespective of the material composition.

First, the length of the cantilever was varied from 50 to 90 µm, for a fixed arbitrary width and height of 10 and 20 µm, respectively. It was observed that the bending angle of the cantilever increased with the length of the beam (Figure 5A,B, Movie S3, Table S3). For a cantilever length of 50 µm, the overall average bending angle was 10.9 ± 2.3° (*n* = 139). Whereas for a cantilever length of 80 µm, the overall average bending angle was 17.7 ± 2.6° (*n* = 139). Hence, increasing the cantilever's length by a factor of 1.6 increased the overall average bending angle by a factor of 1.6. Further increasing the length of the cantilever to 90 µm resulted in larger bending angles; however, cantilevers of this length often failed to remain suspended and instead adhered to the substrate, preventing any mechanical response upon the application of an electric field. Therefore, for all the experiments, a cantilever length of 80 µm was selected. Although high standard deviations were observed for the overall bending angle, they do not indicate inconsistencies in the trend. Instead, they reflect the systematic differences in the bending angles of the cantilevers when bending toward the right (positive values) versus left (negative values). These discrepancies can be caused by the deformations of the cantilever's beam (e.g., twisting) occurring during post‐fabrication processes, and misalignment of the micro‐cantilevers relative to the electric field. Such misalignments include positioning errors, where the micro‐cantilevers are not placed exactly at the midpoint between the cathode (−) and the anode (+), given the large inter‐electrode distance (d_A‐C_ = 15 mm) compared to the cantilever scale, or angular deviations, where the cantilever length is not oriented perpendicular to the electric field.

**FIGURE 5:**
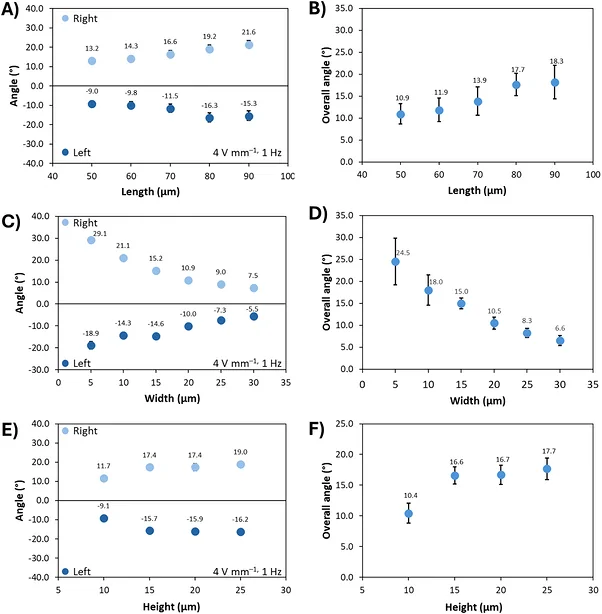
Effect of the geometry of the beam on the electro‐actuation of the p(AA) micro‐cantilevers. (A) Influence of the beam's length on the bending angles of the micro‐cantilevers, and (B) the corresponding overall bending angle. (C) Effect of the beam's width on the bending angles of the micro‐cantilevers, and (D) the corresponding overall bending angle. (E) Impact of the beam's height on the bending angles of the micro‐cantilevers, and (F) the corresponding overall bending angle.

Next, the width of the cantilever was varied from 5 to 30 µm, for a fixed arbitrary height of 20 µm and an optimal length of 80 µm. The bending angle of the cantilever decreased with increasing cantilever width (Figure 5C,D, Movie S3, Table S4). A cantilever width of 5 µm, yielded an overall average bending angle of 24.5 ± 5.3° (*n* = 148), whereas for a cantilever width of 30 µm, the overall average bending angle was 6.6 ± 1.1° (*n* = 133). Hence, increasing the cantilever's width by a factor of 6 decreased the overall average bending angle by a factor of 3.7. Although higher bending angles were recorded for 5 µm width cantilever beams, those beams were demonstrated to be very fragile. For practicality and reproducibility purposes, the 10 µm width was selected as it offered the best compromise between cantilever performance and robustness. These two sets of experiments demonstrated that the bending angle of the cantilever increased with the aspect ratio, which is in agreement with predictions of the three‐point mechanical bending model proposed by Shiga and coworkers [63, 96].

Finally, the height of the cantilever was varied from 10 to 25 µm, for the previously optimized length and width of 80 and 10 µm, respectively. The bending angle of the cantilever increased with height (Figure 5E, F, Movie S3, Table S5). For a cantilever height of 10 µm, the overall average bending angle was 10.4 ± 1.6° (*n* = 149), whereas for a cantilever height of 25 µm, an overall average bending angle of 17.7 ± 1.8° (*n* = 135) was measured. Hence, increasing the cantilever's height by a factor of 2.5 increased the overall average bending angle by a factor of 1.7. Similar to the other experiment related to the geometry optimization, although higher bending angles were recorded for the 25 µm height cantilevers, a height of 20 µm was chosen for robustness (e.g., twisting of the beam occurred often when using a cantilever height of 25 µm) and performance.

Based on these results, it was determined that the optimal dimensions of the micro‐cantilevers developed in this work were 80 × 10 × 20 µm^3^. This configuration provided an effective balance between achieving high bending angles, maintaining geometric stability, and ensuring reproducibility. Therefore, this micro‐cantilever geometry was standardized across all subsequent photoresists and experiments, ensuring reliable and systematic comparisons of their electro‐actuation properties.

### 2.4 Effect of Experimental Conditions on Micro‐Cantilever Actuation

The optimal geometrical parameters (80 × 10 × 20 µm^3^) were then applied to fabricate micro‐cantilevers using the other photoresist solutions to investigate the influence of various experimental parameters such as electric field, electrolyte's ionic strength and pH, on the electro‐actuation behavior of the micro‐cantilevers. By maintaining consistent geometry across different materials, in contrast to the previous section, the results of this study are not only influenced by mechanical factors but also by the intrinsic properties of the polymeric network forming the micro‐cantilevers (e.g., swelling, stiffness, and charge density). As a result, the study was extended to include all the photoresist solutions used in this work.

First, the effect of the electric field strength on the micro‐cantilevers' actuation was examined under standard experimental conditions (50 mM phosphate buffer solution of pH 7.4) by varying the potential applied to the electrodes, fixed at a constant distance (d_A‐C_ of 10 mm). The micro‐cantilevers fabricated with the three different photoresists (p(AA), p(AMPS), and p(APTAC)) followed the same trend, with an increase in the bending angle with increasing electric field strength (Figure 6A,B, Movie S4, Table S6). Additionally, maximum actuation was achieved by the p(AMPS) cantilevers, which exhibited the highest overall bending angle of 46.7 ± 4.8° (*n* = 1032) using an electric field of 5 V mm^−1^. The second‐highest actuation response was achieved by the p(AA) cantilever with an overall average bending angle of 30.2 ± 3.1° (*n* = 384) at an electric field of 6 V mm^−1^. The p(APTAC) micro‐cantilever displayed the smallest degree of actuation, with a recorded bending angle of 5.7 ± 2.2° (*n* = 557) at 5 V mm^−1^. Overall, a linear relationship between the electric field strength and the overall bending angle of the micro‐cantilevers was observed up to 4 V mm^−1^ for both p(AMPS) and p(APTAC) and up to 6 V mm^−1^ for p(AA). This result has been empirically demonstrated in previous experiments [56, 94, 97, 98, 99, 100], but also theoretically demonstrated by Shiga and co‐workers using a three‐point mechanical bending model [63, 96]. As the electric field increases, the drift velocity of the mobile ions also increases, enhancing the ionic concentration gradient across the hydrogel and within the electric double layer (EDL), and consequently the osmotic pressure difference it experiences [58, 75, 101]. As the electro‐actuation of the hydrogel relies on this electro‐induced osmotic pressure difference, a greater gradient leads to larger bending angles [56, 101, 102]. Although higher bending angles were recorded above 4 V mm^−1^, repeated and prolonged experiments at these electric field strengths resulted in evaporation of the electrolyte solution, leading to an increase in the electrolyte concentration. This change negatively affected the actuation amplitude of the micro‐cantilevers (Figure S19). Additionally, higher electric fields promoted bubble formation, which generated air pockets that disrupted the homogeneous current distribution within the electrolyte solution. Increasing the electric field strength to ≥5 V mm^−1^ can also compromise the reliability of automated actuation tracking (Figure S19E). This effect becomes apparent after approximately 15 actuation cycles (Figure S19E), when the generation of a large number of gas bubbles interfered with the accurate tracking of actuator motion. Nevertheless, renewal of the electrolyte solution (marked in Figure S19E) restored the actuator's performance to that observed at the beginning of the cycle (Figure S19). Therefore, the optimal electric field represents a compromise between maximizing bending amplitude and ensuring practical operation and experimental reproducibility. An electric field of 4 V mm^−1^ was selected for studying the effect of elecrolyte pH and concentration on electro‐actuation, as it provided reliable actuation under milder operating conditions while maintaining consistent performance over the course of the experiment.

**FIGURE 6:**
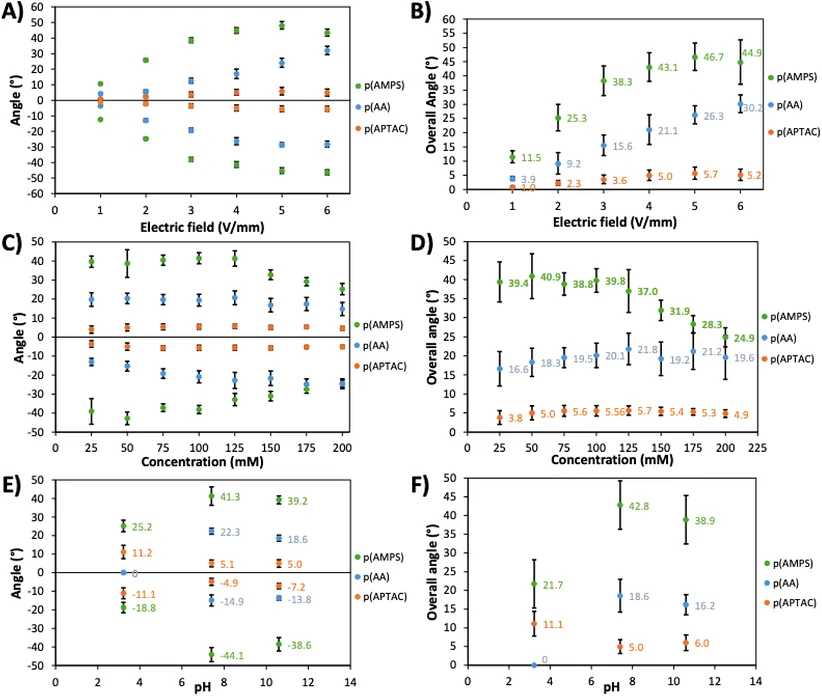
Effect of experimental conditions on the electro‐actuation of the p(AA), p(AMPS), and p(APTAC) micro‐cantilevers. (A) Influence of the electric field strength on the bending angle of the micro‐cantilevers, and (B) the corresponding overall bending angle. (C) Effect of the concentration of the electrolyte solution on the bending angle of the micro‐cantilevers, and (D) the corresponding overall bending angle. (E) Effect of the pH of the electrolyte solution on the bending angle of the micro‐cantilevers, and (F) the corresponding overall bending angle.

It is worth noting that at 1 V mm^−1^, the bending angles of the micro‐cantilevers drop below 12° for all compositions described herein. This contrasts with macro‐scale EAHs, which can continue to actuate at lower electric field strength (i.e., 0.1–0.6 V mm^−1^) [38, 45, 53, 56, 102, 103, 104]. This discrepancy could stem from the difference in Young's moduli between hydrogels fabricated at the macro‐scale using photo‐induced free radical polymerization, and those fabricated using DLW by 2PP. The voxel‐by‐voxel fabrication process inherent to DLW by 2PP requires a high concentration of reactive species within each voxel to initiate free‐radical polymerization, resulting in highly crosslinked polymer networks. Typically, non‐mechanically reinforced EAHs present Young's moduli in the order of kPa [34, 45, 102, 103, 105]. For instance, O'Grady et al. reported that the Young's modulus of poly(acrylate) hydrogels was 10.5 ± 1.7 kPa in HEPES and Na_2_PO_4_ buffered solution of pH 7.5 [106]. In contrast, the p(AA) hydrogels fabricated in this work using DLW exhibited Young's moduli in the order of ∼ 9–33 MPa. Potential strategies to overcome the trade‐off between fabricating soft microstructures (with low Young's moduli) and maintaining high feature resolution include the incorporation of nanofillers [107], the use of deep eutectic photoresists [108], or the introduction of secondary polymer networks such as PVP [83] or GelMA as in the present work. Accordingly, p(APTAC) microstructures displayed a low Young's modulus of 150 kPa (Figures 3E,F, S12B, and S13B), which is much closer to the values reported for macroscale hydrogels based on similar monomers (100–800 kPa) [45, 103, 109]. Consequently, stiffer hydrogel networks, as is the case of the micro‐cantilevers described herein, require higher osmotic pressure for actuation, achieved by applying higher electric fields.

The effect of the electrolyte's ionic strength was studied upon applying an electric field strength of 4 V mm^−1^. The cantilevers fabricated using either p(AA) or p(APTAC) displayed a relatively stable actuation response across the whole concentration range tested (25 to 200 mM), with a small increase in the bending angle up to 125 mM followed by a small decrease in the bending angle for the higher end of the ionic strength range (Figure 6C,D, Movie S5, Table S7). Cantilevers fabricated using p(AMPS) demonstrated the highest sensitivity toward the ionic strength, with a stable bending angle of ∼ 40° in the range of 25–125 mM, followed by a large decrease in the extent of actuation to reach a bending angle of 24.9 ± 2.5° (*n* = 582), when the electrolyte concentration was 200 mM. These results align with the dynamic enrichment and depletion mechanism commonly used to describe the electro‐actuation mechanism of EAHs. As previously discussed, the actuation of the hydrogel is driven by the electro‐induced difference of osmotic pressure between the two interfaces of the hydrogel (Δπ_*ion*_). This osmotic pressure (π_*ion*_) relies on the difference in the concentration of mobile ions inside ($C_{i}^{g}$) and outside the hydrogel ($C_{i}^{s}$), as described by the following equations Equations (3) and (4) [45, 61, 62, 63]:

(3) $$\pi_{ion}=R\cdot T\cdot \sum_{i}C_{i}^{g}-C_{i}^{s}$$

(4) $$\Delta \pi_{ion}=\pi_{anode}-\pi_{cathode}$$

where π_*ion*_ is the osmotic pressure due to ions at a given location (Pa), *R* is the gas constant (J K^−1^ mol^−1^), *T* is the temperature (K), $C_{i}^{g}$ and $C_{i}^{s}$ are the concentration of ions in the gel and the solution (mol L^−1^), respectively. Δπ_*ion*_ is the osmotic pressure difference, π_*anode*_ and π_*cathode*_ are the osmotic pressures at the hydrogel interface facing the anode (+) and the cathode (−), respectively.

Therefore, an increase in $C_{i}^{s}$ results in a smaller osmotic pressure between the two hydrogels’ interfaces due to the concentration of mobile ions in solution being closer to that in the hydrogel, resulting in a smaller actuation response.

Finally, the effect of the pH of the electrolyte solution upon applying an electric field strength of 4 V mm^−1^ was investigated. In addition to the electro‐actuation experiments conducted in a 50 mM phosphate buffer solution of pH 7.4, the electro‐actuation of the micro‐cantilevers was also tested in a 50 mM potassium phthalate (KHP) buffer solution of pH 3.2, and in a 50 mM sodium bicarbonate (NaHCO_3_) buffer solution of pH 10.6. As expected, no mechanical response upon applying an electric field was observed for the p(AA) micro‐cantilevers in the 50 mM KHP buffer solution of pH 3.2, in contrast to the p(AMPS) and p(APTAC) micro‐cantilevers (Figure 6E,F, Table S8). The p(AMPS) cantilever displayed a large decrease in actuation at pH 3.2 with an overall bending angle of 21.7 ± 4.3° (*n* = 568), while the p(APTAC) cantilever had an increase in overall bending angle of 11.1 ± 3.3°, compared to pH 7.4 conditions. The overall average bending angle of the p(AA) micro‐cantilevers in the buffer solutions of pH 7.4 and pH 10.6 were 18.6 ± 4.4° (*n* = 411) and 16.2 ± 2.7° (*n* = 528), respectively. For the p(AMPS) micro‐cantilevers, the overall average bending angles recorded were 42.8 ± 4.6° (*n* = 587), and 38.9 ± 3.0° (*n* = 601), whereas for the p(APTAC) micro‐cantilevers the overall average bending angles recorded were 5.0 ± 1.8° (*n* = 591), and 6.0 ± 2.1° (*n* = 562) in the buffer solutions of pH 7.4 and pH 10.6, respectively. Therefore, p(AMPS) and p(APTAC) displayed opposite actuation trends as a function of pH.

### 2.5 Multi‐Material EAH Micro‐Actuators

Following the optimization and characterization of micro‐cantilever structures, multi‐material microstructures fabrication via DLW by 2PP was explored using combinations of the three photoresists developed. The multi‐material fabrication would not only allow for further expansion of the complexity of the structures achievable using 2PP, but also for the creation of more complex micro‐actuators. As a proof‐of‐concept, two of the previously studied photoresists were paired for the fabrication of a gripper‐like structure. The structures were prepared using a three‐step fabrication process (Experimental Section, Supporting Information). First, a base (140 × 24 × 40 µm^3^) was fabricated using the IP‐L resin, with a slicing and hatching distance of 0.3 µm, at 30 mW and 10 mm s^−1^. In the second step, a suspended cantilever (80 × 20 × 10 µm^3^) was connected to the base using one of the GelMA‐based photoresists. The cantilever was fabricated at 27.5 mW and 1 mm s^−1^ when using the AMPS‐photoresist, or at 37.5 mW and 2.5 mm s^−1^ when using the APTAC‐photoresist. In the last step, a second suspended cantilever (80 × 20 × 10 µm^3^) was connected to the base at its opposite end, using either one of the GelMA‐based photoresists or the AA‐photoresist at 30 mW and 10 mm s^−1^.

The gripper‐like structure composed of the two GelMA‐based photoresists was fabricated to create a structure in which individual structural elements displayed bending in opposite directions (Figures 7Ai‐iii and Bi‐iii). The structure was then tested in electrolyte solutions of different pH values, using an electric field of 4 V mm^−1^ at 1 Hz (Movie S6). The micro‐cantilevers displayed a fast electro‐actuation over the entire pH range tested due to the charge carried by the p(APTAC) and p(AMPS) networks under these pH conditions. At the same time, the micro‐cantilevers bent in opposite directions, with each cantilever bending toward the electrode carrying the same charge as its polymeric network (Figure 7Ai‐iii). The structure also maintained its reversible actuation capability upon reversal of the electric field direction (Figure 7Bi‐iii). As previously described for single material structures, under pH 3.2 conditions, the p(APTAC) micro‐cantilever exhibited visibly increased bending angles, while the p(AMPS) micro‐cantilever showed reduced bending (Figure 7Ai,Bi), compared to pH 7.4 (Figure 7Aii,Bii) or pH 10.6 conditions (Figure 7Aiii,Biii). These changes are consistent with the increased contribution of positive charges introduced by the GelMA‐based polymeric matrix at pH < isoelectric point, which affects the overall charge distribution and, consequently, the electro‐actuation behavior.

**FIGURE 7:**
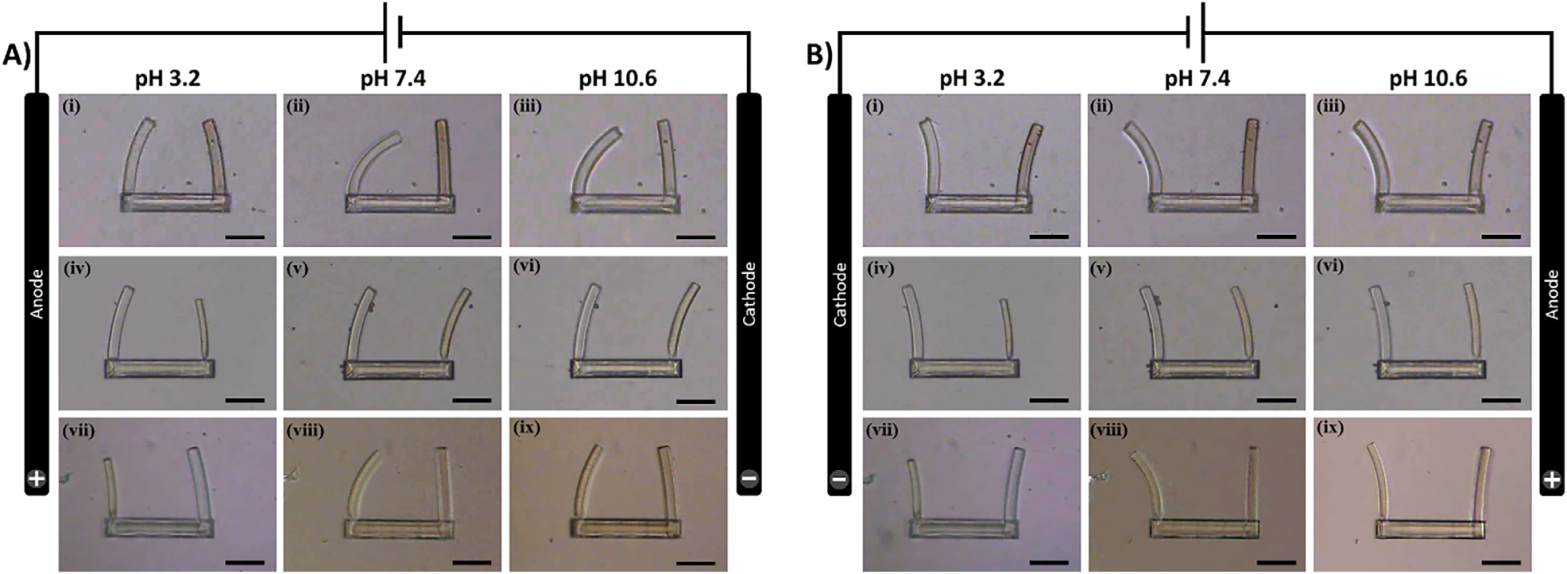
Microscopy images of p(AMPS)∖p(APTAC) (i–iii), p(AMPS)∖p(AA) (iv–vi), and p(AA)∖p(APTAC) (vii–ix) multimaterial structures in electrolyte solutions of pH 3.2 (i, iv, vii), pH 7.4 (ii, v, viii), and pH 10.6 (iii, vi, ix) upon applying (A) an electric field (4 V mm^−1,^ 1 Hz) and (B) upon reversing the direction of the electric field (4 V mm^−1^). The scale bars represent 50 µm.

The two anionic AA‐ and AMPS‐photoresists were also combined to leverage the pH‐dependent electro‐actuation of the AA‐photoresist, hence creating a structure in which individual structural elements could be activated depending on the pH of the aqueous electrolyte (Movie S6). The structures were first tested in solution at pH 3.2, in response to the electric field (4 V mm^−1^, 1 Hz), where the p(AMPS) micro‐cantilever displayed a fast actuation toward the cathode (−) (Figure 7Aiv), and switched bending direction upon field reversal (Figure 7Biv), whereas the p(AA) micro‐cantilever did not show any electro‐active behavior due to its uncharged polymeric network under such conditions (Figure 7Aiv and Biv). However, the electro‐actuation of the p(AA) micro‐cantilever could be reactivated when the structures were exposed to electrolyte solutions of pH above its p*K_a_*. Both micro‐cantilevers demonstrated electro‐actuation toward the cathode in response to the electric field (4 V mm^−1^, 1 Hz) when tested at pH 7.4 (Figure 7Av) and pH 10.6 (Figure 7Avi), and displayed reversibility upon switching the polarity of the electrodes (Figure 7Bv, vi).

Finally, the AA‐ and APTAC‐photoresists were used to create a gripper that displayed bending in opposite directions and a pH‐dependent electro‐actuation (Movie S6). Similarly, the p(AA) micro‐cantilevers did not show any actuation at pH 3.2 (Figure 7Avii,Bvii) owed to its non‐ionized state, whereas the p(APTAC) micro‐cantilever displayed a fast bending toward the anode (+) (Figure 7Avii) and reverted its direction of bending upon reversal of the electric field (Figure 7Bvii). Once exposed to pH 7.4 and 10.6, the structure presented individual structural elements bending in different directions, with the p(AA) micro‐cantilever bending toward the anode (+) and the p(APTAC) micro‐cantilever bending toward the cathode (−) (Figure 7Aviii, ix), as predicted by the charge carried by their respective polymeric network, and their reversibility capabilities were also preserved upon changes in the electric field direction (Figure 7Bviii, ix).

These proof‐of‐concept structures demonstrated the realization of electrically responsive microstructures in which the electro‐actuation of each element can be tuned based on the material's chemistry and the pH of the aqueous electrolyte. Specifically, the combination of opposite and permanently charged monomers, such as AMPS and APTAC, enables the fabrication of microstructures whose electro‐actuation direction can be controlled not only by the direction of the electric field but also by the charge of the monomer. This approach allows for the realization of multidirectional actuation using a single electric field orientation and could pave the way for the integration of independently addressable micro‐actuators within a single platform operating over a broad pH range (3.2–10.6). The combination of a pH‐responsive monomer such as AA with a permanently charged monomer (i.e APTAC) offers a higher level of functional complexity, in which the electro‐actuation of the individual structural elements can be turned on and off, depending on the local chemical environment. This introduces the possibility of a dual‐responsive or logic behavior, where actuation is triggered only under specific chemical and electrical conditions.

### 2.6 4D Actuation

The realization of micro‐actuators exhibiting different modes of actuation beyond simple unidirectional bending was also explored. These studies employed the AMPS‐photoresist given its superior electro‐active performance in the series.

The first design explored a twisting motion through the fabrication of a non‐responsive IP‐S base (50 × 50 × 8 µm^3^), on which a vertical pillar (10 × 40 × 120 µm^3^) was positioned (Figure 8). The twisting motion was executed through the bending of the pillar with a mechanical constraint along an oblique direction [110, 111, 112]. By taking advantage of the freedom of fabrication offered by 2PP, an oblique internal section (10 µm thick, 60°) was incorporated using a higher laser power (50 mW) into the vertical pillar fabricated at a lower laser power (22.5 mW). Thus, increasing the local crosslinking density (Figure 8A) resulted in a stiffer section mechanically constraining the structure along the oblique axis (Figure 8A). Consequently, the top‐left edge is expected to bend more than the top‐right edge, generating torsional forces that twist the pillar around the *z*‐axis (Figure 8B). As predicted, the pillar demonstrated a torsional motion around the *z*‐axis with a twisting angle, γ, of 22 ± 1° (*n* = 25). When the cathode (−) was positioned on the left side of the pillar, the pillar twisted in a clockwise direction (Figure 8Cii, Movie S7). Conversely, when the polarity was switched, and the cathode (−) was positioned on the right side of the pillar, the pillar twisted in an anticlockwise direction (Figure 8Ciii).

**FIGURE 8:**
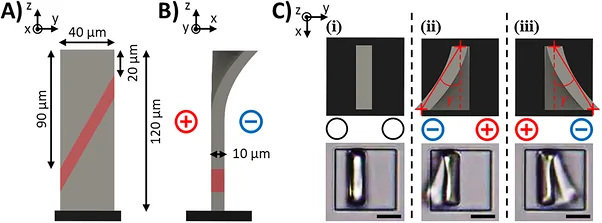
(A) Frontal view of vertical pillar 3D model. (B) Profile view of the vertical pillar 3D model in response to an applied electric field. (C) Top view of the 3D model (*Top*) and the fabricated vertical micro‐pillar (*Bottom*), (i) before applying an electric field, (ii) after applying an electric field (4 V mm^−1^, 1 Hz), and (iii) upon reversing the polarity of the electrodes (⊕: anode; ⊖: cathode; o: neutral). The red section of the pillar represents the section fabricated at a higher laser power. The scale bars represent 20 µm.

The second design explored the capability of the electrical setup to spatially modulate the electric field, enabling electro‐actuation with greater degrees of freedom (DOF). Most previous works on EAHs relied on uniform field modulation, which does not fully exploit the level of control offered by electric fields and only enabled unidirectional bending (1 DOF). Here, a four‐electrode system was used to control the electric field direction in a 2D plane to induce 360° bending of a pillar around its base. The structure fabricated consisted of a non‐responsive IP‐S base (50 × 50 × 8 µm^3^), in which a vertical square pillar (40 × 40 × 120 µm^3^) was embedded (Figure 9A). The base of the pillar consisted of a stable cross‐shaped structure (40 × 40 × 40 µm^3^) to provide structural integrity, while the top of the pillar was fabricated with a high aspect ratio (10 × 10 × 80 µm^3^). Electro‐actuation was performed using a programmable switch that sequentially addressed pairs of opposite or neighboring electrodes, with a frequency of 1 Hz, in a pattern that steered the pillar in a 360^○^ anticlockwise circular path. The electric field applied was 4 V mm^−1^, while the inter‐electrode distance between opposite Pt electrodes was 15 mm.

**FIGURE 9:**
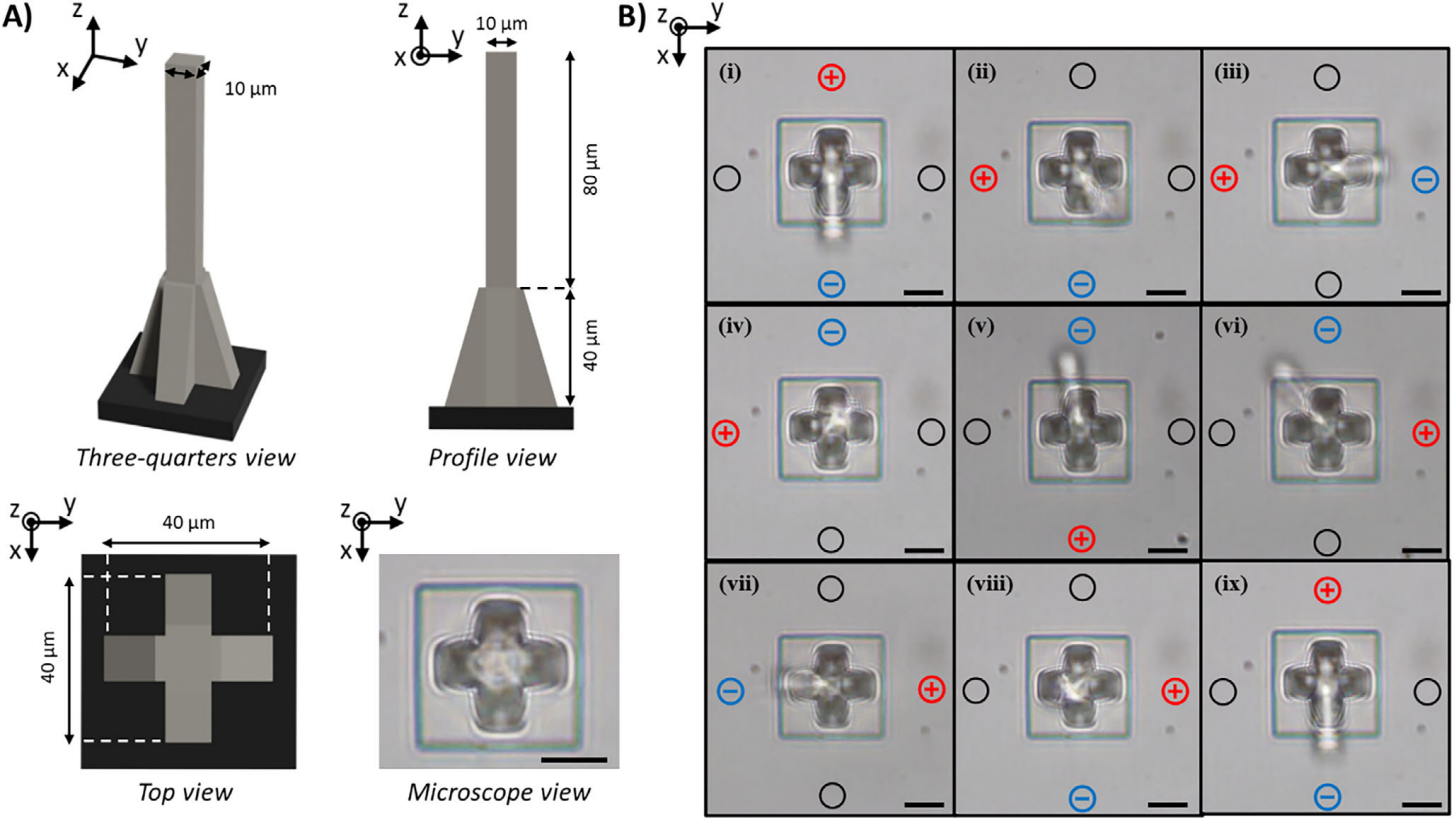
(A) 3D model of the square pillar shown in three‐quarters view (*Top Left*), profile view (*Top Right*), top view (*Bottom Left*), and a top‐view microscopy image (*Bottom Right*). (B) Sequential top‐view microscopy images of the square pillar during electro‐actuation (4 V mm^−1^, 1 Hz). The pillar describes a circular path in an anticlockwise direction in 45° increments from (i) to (ix). The four circles represent the positions of electrodes along with their polarities (⊕: anode; ⊖: cathode; o: neutral). The scale bars represent 20 µm.

Top view microscopy (Figure 9B, Movie S7) showed the pillar deflecting toward the cathode (−) and thereby traversing a circular path from (i) to (ix) in 45^○^ increments, starting and finishing with the pillar actuating downwards. This study demonstrated that leveraging the spatial modulation of the electric field increased the structure's DOF to 2. The pillar responded to the electric field with a response time of 100 ±  50 ms (*n* = 12), similar to the actuation response achieved for the cantilevers. The approximate radius of the traversed path achieved with an electric field of 4 V mm^−1^ was 35 ±  5 µm (*n* = 20). The large uncertainty is likely caused by the square shape of the pillar, whose thickness is not symmetric in all orientations and therefore gives rise to varied deflections. Future integration of on‐chip microelectrodes would enable precise positioning of the electrodes around the actuator and localized modulation of the electric field, thereby improving steering accuracy and expanding the number of accessible degrees of freedom (DOFs). Furthermore, reducing the distance between the microstructure and the electrodes would substantially decrease the electric field required for actuation. Established fabrication methods for integrated microelectrodes include photolithography [75, 113, 114, 115], stereolithography [116, 117, 118], or electrical discharge machining [119].

These two studies demonstrated that leveraging the ability of 2PP to tune the spatial distribution and mechanical properties of the printed material, and the capability of the electrical setup to spatially modulate the electric field, are effective strategies to empower EAH with actuation modes beyond unidirectional bending. These strategies could be further explored to create more complex patterned structures and sophisticated modes of motion, as well as by increasing the number of electrodes or modifying electrode designs to generate alternative spatial configurations of non‐uniform electric fields.

## 3 Conclusion

The work reported herein represents the first microscale example of GelMA‐based hydrogel electro‐actuators. These displayed rapid response times (∼ 200 ms) and large bending angles (up to 46.7 ± 4.8° at 5 V mm^−1^). Notably they also demonstrated actuation of up to 11.5 ± 2.1° in response to electric fields as low as 1 V mm^−1^. This was achieved using polyelectrolyte hydrogel micro‐structures fabricated by direct laser writing (DLW) via two‐photon polymerization (2PP) with photoresist solutions based on permanently charged or ionizable monomers. Electro‐actuation was demonstrated using micro‐cantilever structures immersed in an electrolyte solution, quantified by measuring their bending angles in response to the applied electric field, and systematically characterized with respect to the hydrogel chemical composition. The design of the micro‐cantilevers was geometrically optimized using AA‐photoresist, while the electro‐actuation of the p(AA)‐, p(AMPS)‐, and p(APTAC) micro‐cantilevers was systematically characterized by varying the electrolyte concentration and pH, along with the electric field direction and strength. Results showed that their electro‐actuation was charge‐dependent with micro‐cantilevers responding only when ionized, while their bending direction was directly correlated to their net charge. Increasing the electric field strength led to a linear increase in bending angle, up to an optimal value of 4 or 5 V mm^−1^, beyond which water electrolysis compromises the electro‐actuation performances. Multi‐material electro‐actuators served as proof‐of‐concept structures, highlighting the possibility for integration of independently addressable micro‐actuators within a single platform, achieving a fast, programmable, and environmentally dependent response. The spatial modulation of the fabrication parameters and the electric field applied yielded more complex actuation responses such as twisting motion and 360° steering motion. Thus, highlighting the potential of DLW‐2PP‐based electro‐active actuators for applications where precision, speed, reversibility, and specific motions are critical, such as for microfluidics, soft robotics, and sensors.

## 4 Experimental Section

### 4.1 Materials and Reagents

Acrylic acid (AA), 2‐acrylamido‐2‐methyl‐1‐propanesulfonic acid (AMPS), (3‐acrylamidopropyl)trimethylammonium chloride (APTAC), poly(ethylene glycol) diacrylate M_n_ = 700 g mol^−1^ (PEGDA700), ethoxylated trimethylolpropane triacrylate M_n_ = 956 g mol^−1^ (TMPET956), *N,N*‐methylenebis(acrylamide) (MBIS), 3‐(trimethoxysilyl)propyl methacrylate, 7‐diethylamino‐3‐thenoylcoumarin (DEATC), and lithium phenyl‐2,4,6‐trimethylbenzoylphosphinate (LAP) were purchased from Sigma‐Aldrich Ireland Ltd. Gelatin methacrylate was synthesized from gelatin (type A 175 bloom from porcine skin) following a literature procedure [120]. Acetic acid, sodium hydroxide (NaOH), hydrochloric acid (HCl, 37%), potassium dihydrogen phosphate (KH_2_PO_4_), and potassium hydrogen phthalate (0.2 M, Reagecon) were also purchased from Sigma‐Aldrich Ireland Ltd, while sodium bicarbonate (NaHCO_3_) was purchased from Fluorochem EU Ltd. All solvents, including acetone, ethanol (EtOH), methanol (MeOH), isopropyl alcohol (IPA), dimethylsulfoxide (DMSO), and propylene glycol methyl ether acetate (PGMEA), were high performance liquid chromatography grade (HPLC) and purchased from Sigma‐Aldrich Ireland Ltd and used without further purification. Large microscope cover glasses (Ø = 30 mm, thickness ∼ 0.16–0.19 mm) and small microscope cover glasses (Ø = 12 mm, thickness 170 ± 5 µm; no. 1.5) were purchased from Carl Stuart Ltd. The epoxy glue used was purchased from Gorilla Glue Europe Ltd. The multi‐range DC power supply Model 9201 60 V/10 A/200 W was purchased from BK Precision and the Pt electrodes were purchased from Sigma‐Aldrich Ireland Ltd. The DLP printers Anycubic Photon Mono and Photon S, and the different colored resins were purchased from ANYCUBIC Technology Co. Ltd.

### 4.2 Photoresists Preparation

The photoresist solutions were obtained by dissolving the appropriate monomer and crosslinker in the corresponding solvent, before adding the photoinitiator. The mixture was then left stirring overnight at 25°C before use for the p(AA) photoresist and at 40°C for the p(APTAC) and p(AMPS) photoresists. The exact compositions of the optimized photoresists used in this study, based on AA (Table 1), AMPS (Table 2) and APTAC monomers (Table 3), are detailed above.

### 4.3 Silanization of the Slides

Prior to micro‐fabrication via 2PP, the glass substrates on which the 3D structures were fabricated were treated as follows. Microscope cover glass slides (Ø = 30 mm) were cleaned by successive immersions in acetone, isopropanol, ethanol, methanol, and DI water, before drying under nitrogen. The slides were then exposed to UV Ozone for 20 min (Ossila, United Kingdom), and subsequently immersed in a solution of 3‐(trimethoxysilyl)propyl methacrylate (3 vol.% in ethanol with 0.1 vol.% acetic acid). After 1 h, the slides were taken out of the silanization solution, rinsed with ethanol, and then dried under nitrogen. Finally, the slides were cured in the oven at 60°C for 3 h.

### 4.4 Fabrication of the EAH Millimeter‐Sized Structures

The macro‐scale cantilever structures were designed using a computer‐aided‐design software (CAD), such as Blender 2.90, before being digitally sliced using the Photon Workshop V2.124 RC7 software from Anycubic. The 3D structures were then fabricated using an Anycubic 3D printer Photon S and following the printed parameters listed in Table S1. After fabrication, the structure was washed in an ethanol bath for 6 min before detaching it from the printing platform, and cured for a further 6 min, using the Anycubic Wash & Cure 1 machine. Finally, the 3D structure was hydrated in a phosphate buffer solution (0.05 M, pH 7.4) over 24 h.

### 4.5 3D Design of Microstructures

The micro‐scale cantilever structures were generated using MATLAB codes created by Dr. Luke Dowling. The other designs, such as Frangipani flower, Hallgrímskirkja church, Owl, Babylon tower, Rose, Venus of Melos, and Water lily, were adapted from files licensed under the Creative Commons—Attribution license. All files are available for download, as detailed in the Supporting Information.

### 4.6 Direct Laser Writing Fabrication

The micro‐scale 3D structures were fabricated on a commercial DLW workstation (Photonic Professional, Nanoscribe GmbH) equipped with a 170 mW femtosecond solid‐state laser (*λ* = 780 nm) that delivers 120 fs pulses with an 80 MHz ± 1 MHz repetition rate. A 63× objective (NA = 1.4, WD = 190 µm) (Zeiss, Plan Apochromat) was used in an oil‐immersion configuration. The position of the sample in the *z*‐axis, and in the *x*‐*y* plane was controlled using a piezo translation stage, and a galvo stage, respectively.

To prepare the sample, the desired photoresist solution was added by capillarity into a home‐made cell consisting of two slides (bottom slide silanized (Ø = 30 mm) and top slide non‐treated (Ø = 12 mm)) attached by a pressure‐sensitive adhesive (PSA) well (15 × 5 × 0.1 mm^3^) cut using a plotter (Cricut Maker 3) (Figure S15A). The cell was flipped, and a drop of oil (Zeiss Immersol 518F) was added in the center of the other face of the silanized slide (Figure 1A). The DLW parameters used for successful fabrication using the different photoresists are available in Figures 2A–C and S1. Once the fabrication was completed, the structures were developed in IPA for 15 min when using the AA‐photoresist, or developed in deionized water at 40°C for 2 min followed by IPA for 2 min when using the GelMA‐based photoresists, to remove the unpolymerized photoresist, and dried under nitrogen.

The fabrication of the multi‐material structures was performed following a three‐step printing process, described in more detail in the Supporting Information (Figure S15). First, a sample was prepared following the aforementioned procedure using the commercially available resin IP‐L (Nanoscribe) to fabricate the base of the structures. Marking lines were made on the glass slide to assist with alignment. Once the fabrication was completed, the structures were developed in PGMEA for 10 min, followed by IPA for 2 min to remove the unpolymerized photoresist, and dried under nitrogen. In the second step, the same slide was used and prepared following the same procedure using one of the GelMA‐based photoresists. Once the fabrication was completed, the structures were developed in deionized water at 40°C for 2 min followed by IPA for 2 min to remove the unpolymerized photoresist, and dried under nitrogen. Finally, the same slide was used and prepared following the same procedure using the AA‐photoresist or one of the other GelMA‐based photoresists. The final structure was subsequently developed in IPA for 15 min when using the AA‐photoresist, or developed in deionized water at 40°C for 2 min followed by IPA for 2 min when using the GelMA‐based photoresists, to remove the unpolymerized photoresist, and dried under nitrogen.

For the fabrication of the twisting pillar and the 360° steerable pillar, a similar protocol was followed using a two‐step printing process. In this case, however, the base of the pillars was fabricated using IP‐S (Nanoscribe).

### 4.7 Electro‐Actuation of the Structures

The electro‐actuation experiments of the mm‐sized cantilevers were carried out in a 3D printed channel filled with phosphate buffer solution (0.05 M, pH 7.4) containing the macro‐scale cantilever. Platinum electrodes were then immersed in the electrolyte solution and connected to a custom‐made polarity switch controller, which was then connected to the power source. The distance between the electrodes was maintained at a constant distance of 10 mm, and a voltage of 60 V was applied. The use of a light box was necessary to keep homogeneous lighting conditions during the whole duration of the experiment to ensure good contrast for the MATLAB program used to process the videos. All the videos of the macro‐scale cantilever were recorded using a DinoLite AM4115T‐CFVW digital microscope at 15× magnification.

For the different electro‐actuation experiments of the micro‐cantilevers, a 3D printed channel was attached to the glass substrate containing the microstructures using a commercially available Gorilla epoxy glue (Figure S16). The 3D channel was then filled with the electrolyte solution, phosphate buffer (0.05 M, pH 7.4), unless otherwise specified. Platinum electrodes were immersed in the electrolyte solution and connected to a custom‐made polarity switch controller, which was then connected to the power source. The electric field strength applied was 4 V mm^−1^, unless otherwise specified, whereas the polarity of the electrodes was switched every 1 s using the custom‐made automated switch. An electro‐actuation cycle was completed once the polarity of the electrodes was switched twice.

To carry out the experiments where the electrolyte solution was changed, the electrolyte solution was removed from the channel, and without moving the set‐up, the channel was rinsed several times with DI water, before refilling it with the electrolyte solution to test.

All the videos showing the electro‐induced actuation of the micro‐cantilevers and microstructures fabricated via 2PP were recorded using an Olympus IX81 microscope equipped with a 10× objective and an Orca Flash camera, and further processed using a custom‐made MATLAB program created by Dr. Luke Dowling and Dr. Annaël Sort‐Montenegro, to measure the bending angle of the cantilever over time. The bending angle was obtained by measuring the deflection angle of the cantilever's beam from its initial position as described in more detail in Supporting Information, Bending angle measurements. The skeletonization function used in the MATLAB program was created by Nicholas Howe (2024) [121].

### 4.8 Scanning Electron Microscopy (SEM)

Scanning electron microscopy of the microstructures fabricated via 2PP was carried out using a Zeiss ULTRA Plus with a GEMINI FESEM column (Zeiss, Germany). An accelerating voltage of 5 kV was used under the SE2 mode to acquire all images. The samples were mounted on aluminum stubs with a conductive carbon tape (Ted Pella, USA) and coated with a conductive Pd/Au layer using an Au‐Pd target (57 × 0.1 mm) that was purchased from Ted Pella Inc and a Cressington 208Hr high‐resolution sputter coater, to improve contrast where static charging interfered with the imaging.

### 4.9 Atomic Force Microscopy (AFM)

Atomic force microscopy of the microstructures fabricated via 2PP was carried out in air, in water, in a HCl solution (pH = 2.7), and in a NaOH solution (0.01 M, pH = 12), using a commercial SPM system (MFP‐ 3D, Asylum Research, USA). AFM height images were acquired using the contact mode with 256 lines per scan direction, at a scan rate and angle of 0.4 Hz and 90°, respectively. Data were further processed using Gwyddion AFM software (version 2.6). The topography measurements were performed using an AFM probe (Electrical all‐in‐one, BudgetSensors, Bulgaria) with four different platinum‐coated AFM cantilevers on a single AFM holder chip, in which cantilever B with a tip radius of 25 nm, a resonant frequency of ∼ 300 Hz, and a spring constant of 45 N · m^−1^ was used for topography and modulus measurements. Line profile data were obtained following image postprocessing and plotted using Igor pro‐6.38. Young's modulus measurements were performed in contact mode, using a PPP‐FM probe (Nanosensors) with a tip radius of 25 nm, resonant frequency of ∼ 75 kHz, and a spring constant of 2.8 N · m^−1^. Before carrying out force curves on the sample, the probe was calibrated to obtain the spring constant and deflection InvOLS (inverse optical lever sensitivity) on a glass slide. The modulus values were obtained by fitting the extension segment of the force curve with Hertz model using the Asylum software. The model assumes a conical tip geometry with a half‐angle of 20° and the sample's Poisson's ratio of 0.45. Each Young's modulus value is an average of three continuous force curves measured for all cubes in the array.

## Author Contributions

**Larisa Florea**: conceptualization, investigation, funding acquisition, writing – review and editing, methodology, formal analysis, project administration, data curation, supervision, resources. **Yekaterina Tskhe**: investigation, methodology, validation. **Luke Dowling**: methodology, software. **Žiga Roblek**: investigation, validation, visualization. **Colm Delaney**: writing – review and editing, methodology, investigation. **Annaël Sort‐montenegro**: investigation, writing – original draft, methodology, validation, visualization, formal analysis. **Jason M. Delente**: investigation, writing – original draft, methodology, validation, visualization, formal analysis, writing – review and editing.

## Conflicts of Interest

The authors declare no conflicts of interest.

## Supporting information




**Supporting File 1**: adma74787‐sup‐0001‐SuppMat.pdf.

**Supporting File 2**: adma74787‐sup‐0002‐MovieS1.mp4.

**Supporting File 3**: adma74787‐sup‐0003‐MovieS2.mp4.

**Supporting File 4**: adma74787‐sup‐0004‐MovieS3.mp4.

**Supporting File 5**: adma74787‐sup‐0005‐MovieS4.mp4.

**Supporting File 6**: adma74787‐sup‐0006‐MovieS5.mp4.

**Supporting File 7**: adma74787‐sup‐0007‐MovieS6.mp4.

**Supporting File 8**: adma74787‐sup‐0008‐MovieS7.mp4.

## Data Availability

The data that supports the findings of this study are available in the supplementary material of this article.
