# Supplementary material for: Fast Hydrogel Micro‐Actuators Driven by Electric Fields
Source: Adv Mater. 2026 Sep 5;38(60):e74787. doi: 10.1002/adma.74787 (PMC13712214; doi:10.1002/adma.74787)
Supplement: Supplementary file 1 [file ADMA-38-e74787-s005.pdf]

Supporting Information

Fast Hydrogel Micro-Actuators Driven by Electric Fields

Annaël Sort-Montenegro,[1†] Jason M. Delente,[1†] Žiga Roblek,[1] Yekaterina Tskhe,[1] Luke Dowling,[2] Colm Delaney,[1] Larisa Florea[1]**

[†] These authors contributed equally to this work

[1]School of Chemistry & AMBER, The SFI Research Centre for Advanced Materials and BioEngineering Research, Trinity College Dublin, D02 PN40, Ireland

[2]Department of Mechanical & Manufacturing Engineering, Trinity College Dublin D02 PN40, Ireland

E-mails: delentej@tcd.ie, floreal@tcd.ie

Table of Contents

Optimization of laser dosage for successful DLW-fabrication 2
SEM images 3
RAMAN spectroscopy 5
AFM data 7
Fabrication of micro-cantilevers 11
Multi-material fabrication 12
Electro-actuation of micro-cantilevers 14
  Channels fabrication 14
  Experimental setup 15
  Bending angle measurements 16
  Electro-actuation data 17
  Quantitative cycle-life testing (> 100 cycles) 20
Ion mobility 23
3D design of microstructures 24
Videos 25
  Movie S1. Micro-scale p(AA), p(AMPS), and p(APTAC) micro-cantilevers 25
  Movie S2. Electro-actuation of the p(AA) macro-cantilever 25
  Movie S3. Electro-actuation of p(AA) micro-cantilevers of varied geometries 26
  Movie S4. Electro-actuation of micro-cantilevers under different electric field strengths 26
  Movie S5. Electro-actuation of micro-cantilevers at different electrolyte concentrations 26
  Movie S6. Electro-actuation of multi-material structures 27
  Movie S7. Electro-actuation of 4D actuators 27
References 27

Optimization of laser dosage for successful DLW-fabrication

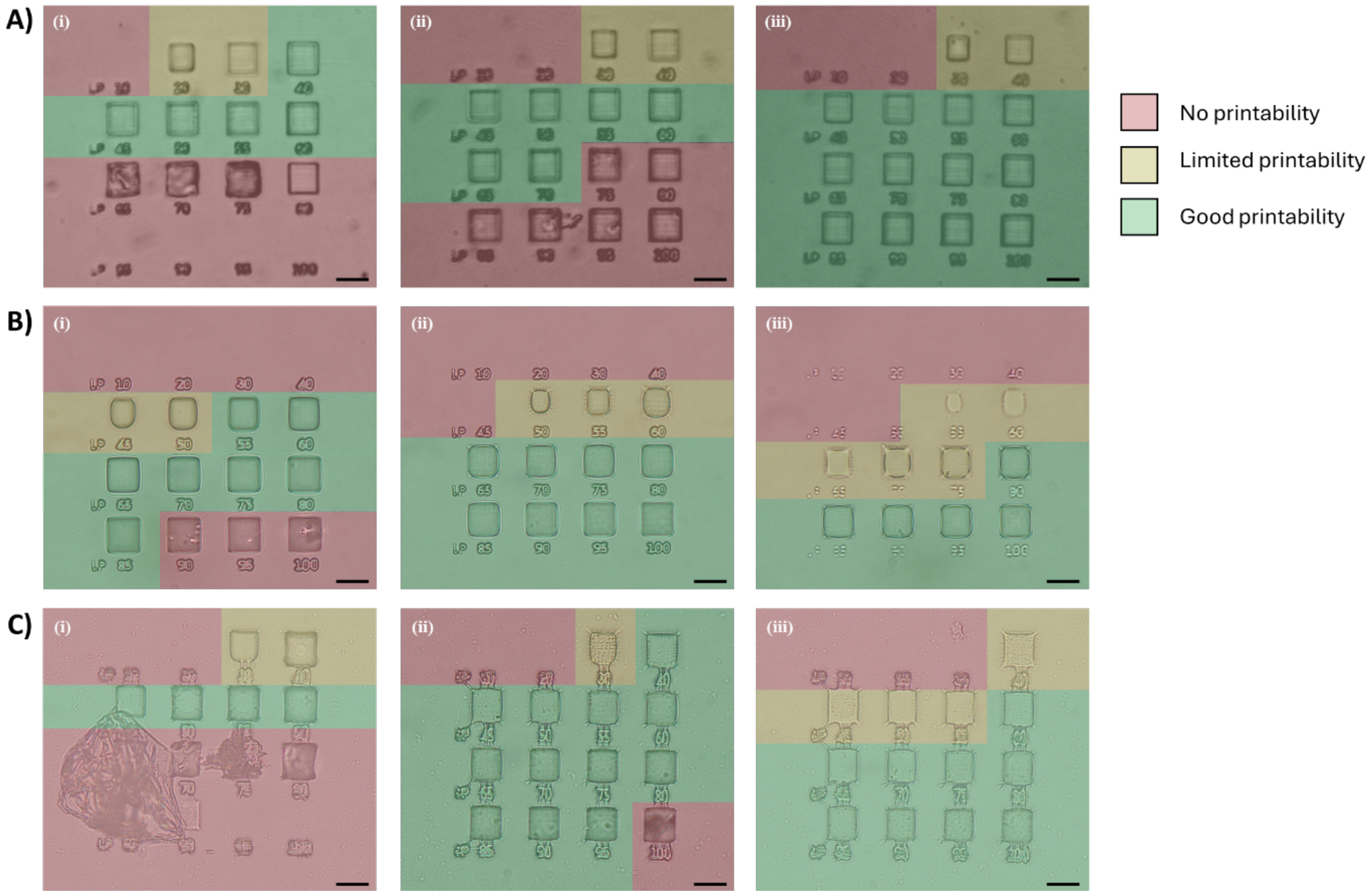

Figure S1. Microscope images of cubes (10×10×4 µm^3) fabricated *via* 2PP using: A) the AA photoresist at a scanning speed of 10 mm s^{-1} and using varying laser power at slicing distances i) 0.1 µm, ii) 0.2 µm, and iii) 0.3 µm; B) the AMPS photoresist at a scanning speed of 1 mm s^{-1} and using varying laser power at slicing distances i) 0.1 µm, ii) 0.2 µm, and iii) 0.3 µm; and C) the APTAC photoresist at a scanning speed of 2.5 mm s^{-1} and using varying laser power at slicing distances i) 0.1 µm, ii) 0.2 µm, and iii) 0.3 µm. The scale bars represent 10 µm. The laser power (LP) was increased incrementally from 10% to 100% (corresponding to 50 mW), as indicated underneath each cube.

SEM images

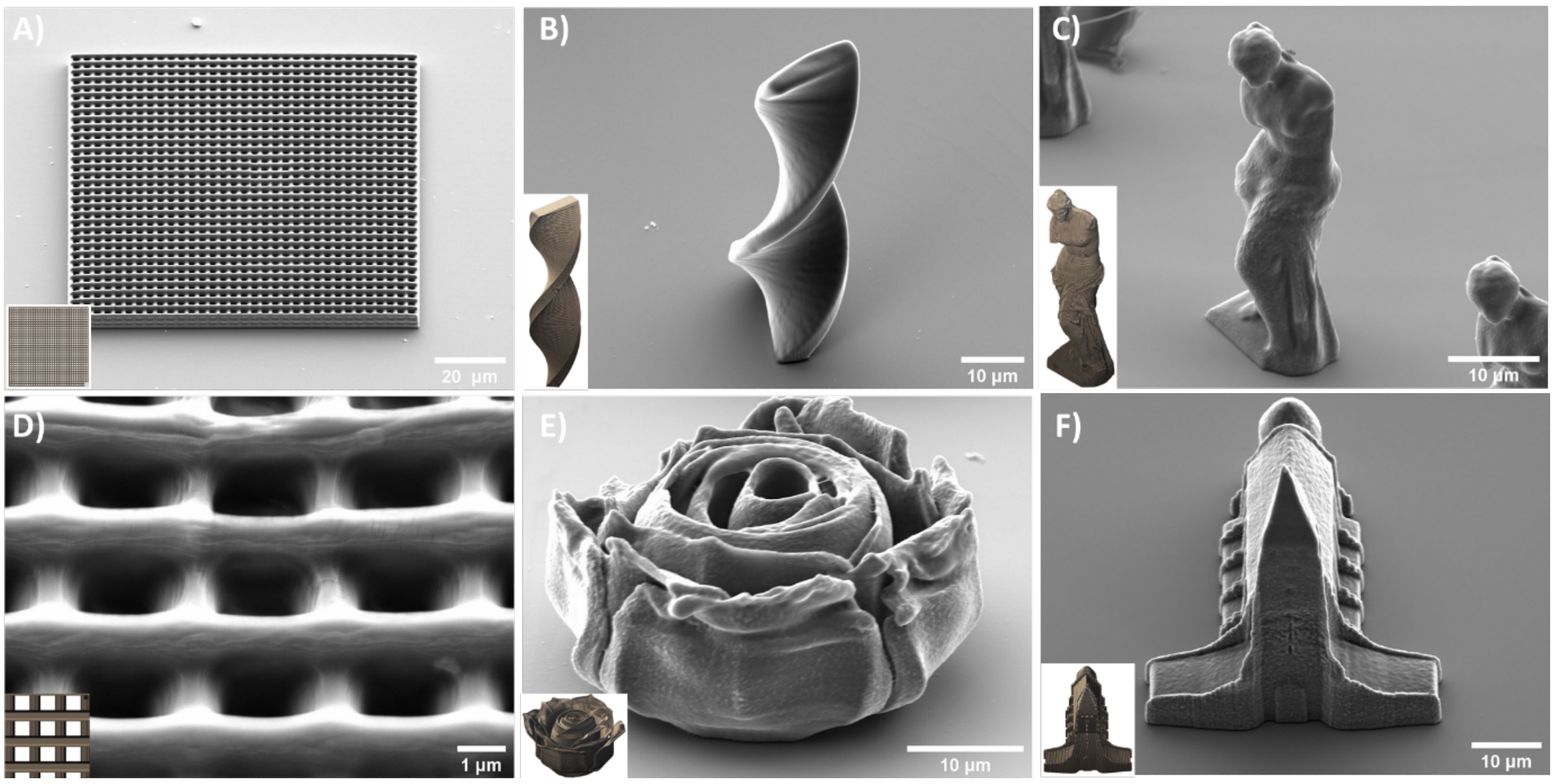

Figure S2. Scanning electron microscopy images of structures fabricated using the AA-photoresist of: A) and D) a 5-layer woodpile (35 mW and 10 mm s^{-1}, tilt 42.2˚), B) a 80×4×20 μm^3 pillar with a 360° twist (30 mW and 10 mm s^{-1}, tilt 38˚), C) Venus of Melos (30 mW and 10 mm s^{-1}, tilt 55˚), E) a rose (20 mW and 10 mm s^{-1}, tilt 55˚), and F) Hallgrímskirkja church (32.5 mW and 10 mm s^{-1}, tilt 42.2˚). All the structures were fabricated with a slicing and hatching distance of 0.1 µm.

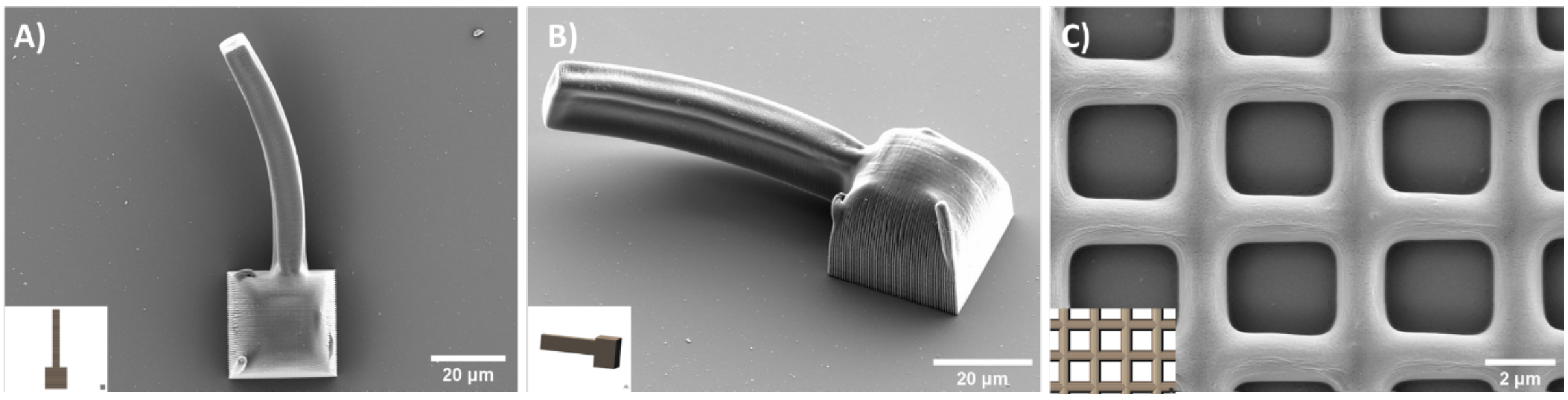

Figure S3. Scanning electron microscopy images of structures fabricated using the AMPS-photoresit of: A) Top view and B) tilted view (40 °) of cantilever fabricated with the base in IP-L (30 mW at 10 mm s^{-1}) and the beam in AMPS (45 mW at 1 mm s^{-1}) with a slicing distance 0.3 µm. C) Grid (50 mW at 1 mm s^{-1}) fabricated with a slicing distance of 0.1 µm. The scale bar represents A) and B) 20 µm, C) 2 µm.

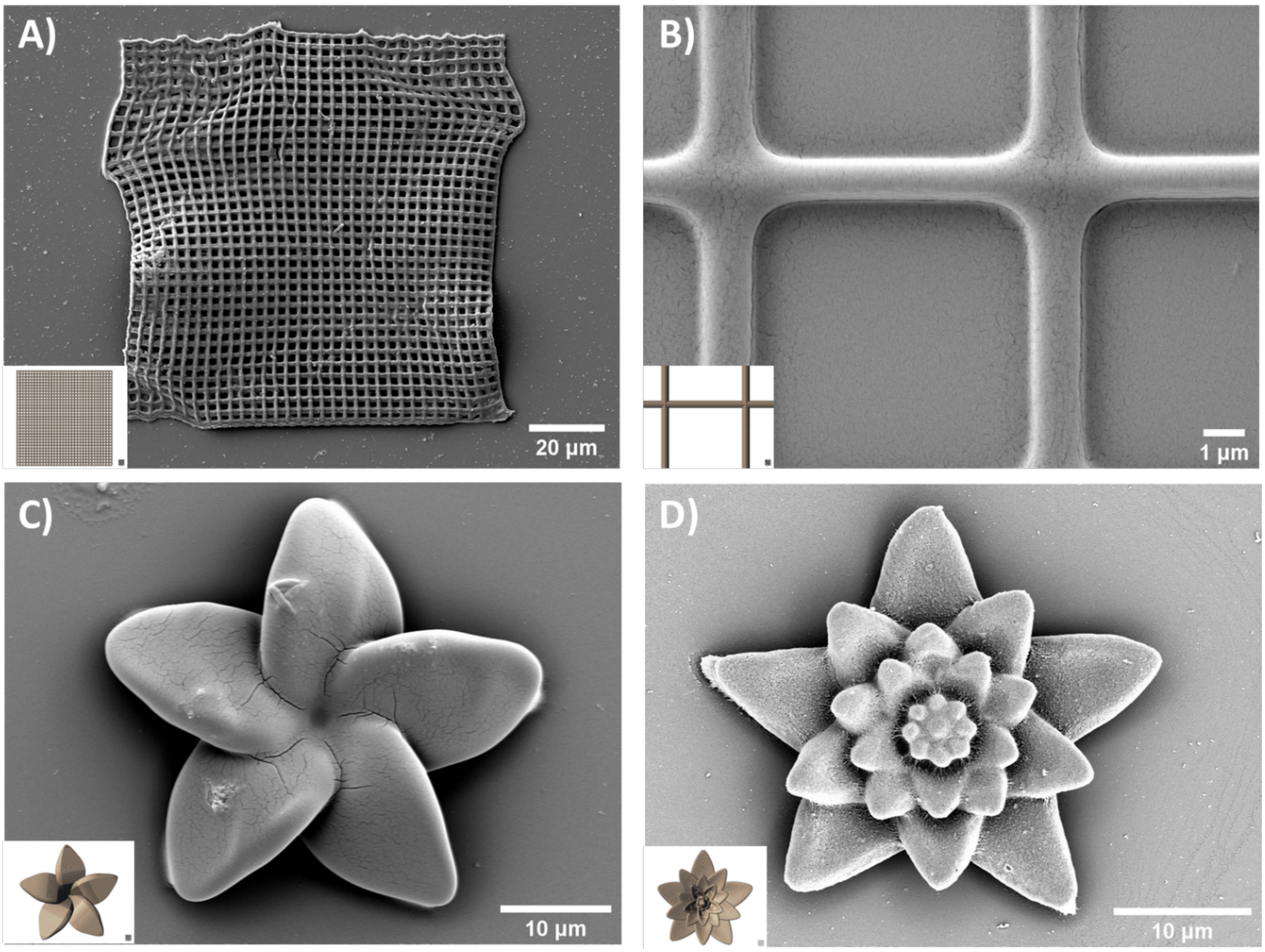

Figure S4. Scanning electron microscopy images of structures fabricated using the APTAC-photoresist. A) Woodpile (30 mW, 10 mm s^{-1}), B) zoomed in grid (25 mW, 2.5 mm s^{-1}), C) plumeria (22.5 mW and 2.5 mm s^{-1}), and D) waterlily (30 mW and 10 mm s^{-1}). All structures were fabricated with a slicing and hatching distance of 0.1 µm. The scale bar represents A) 20 µm, B) 1 µm, C) and D) 10 µm.

RAMAN spectroscopy

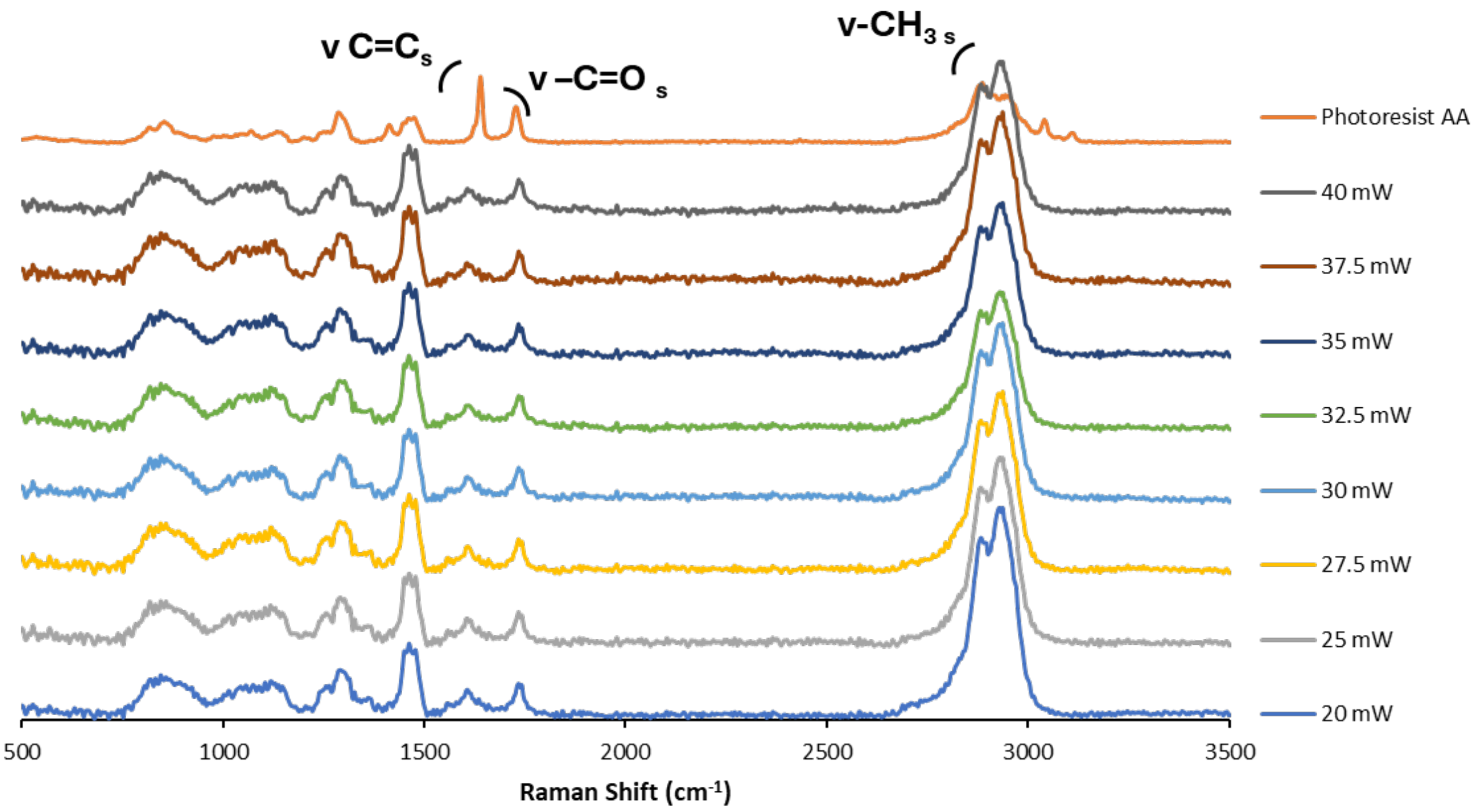

Figure S5. Stacked Raman spectra of the AA photoresist and micro-cubes fabricated at various laser powers.

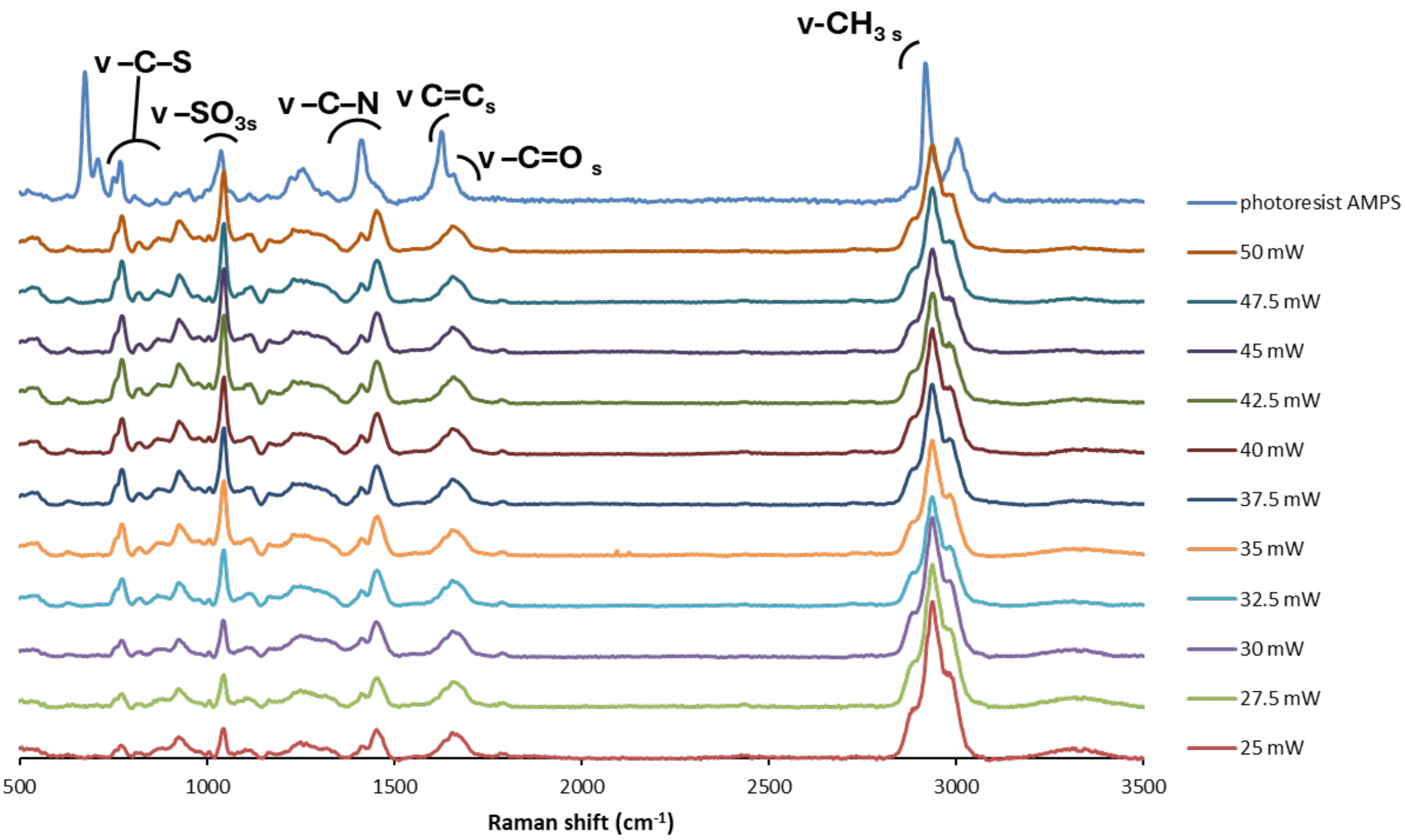

Figure S6. Stacked Raman spectra of the AMPS photoresist and micro-cubes fabricated at various laser powers.

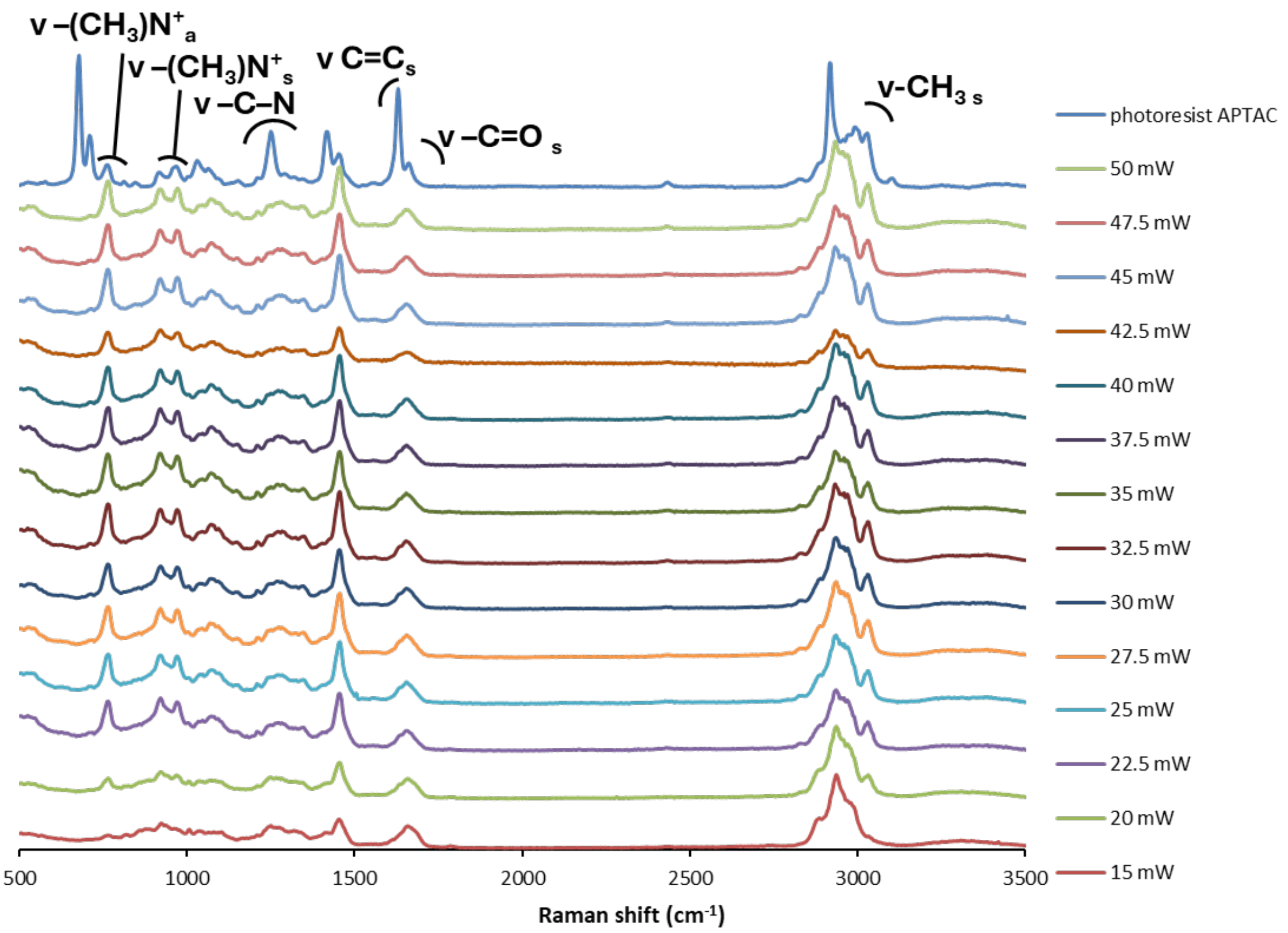

Figure S7. Stacked Raman spectra of the APTAC photoresist and micro-cubes fabricated at various laser powers.

AFM data

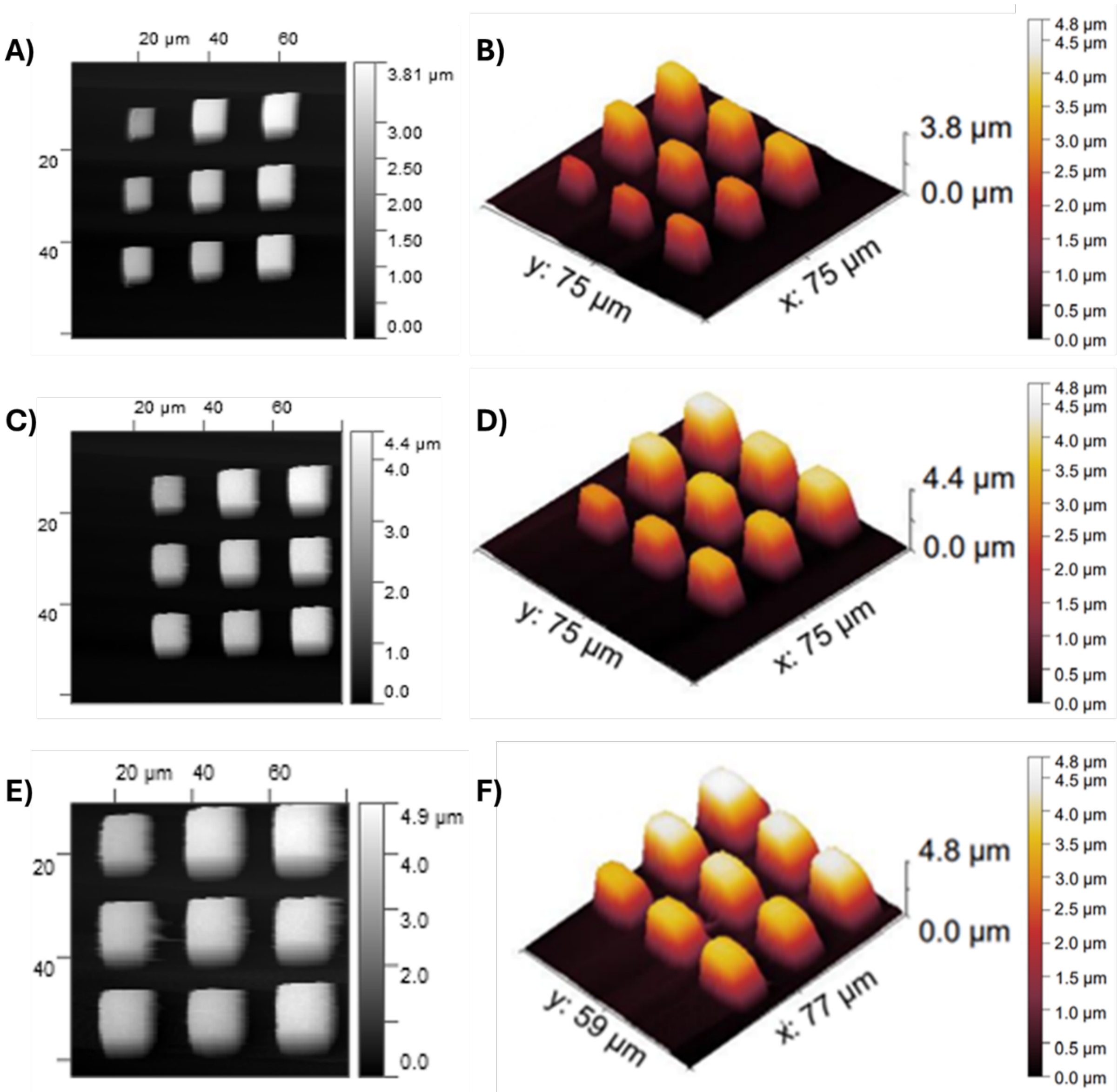

Figure S8. Topography obtained by AFM of p(AA) micro-cubes in their dry state in A) top view and B) 3D; in their hydrated state in HCl solution (pH 2.7) in C) top view and D) 3D; in their hydrated state in a NaOH solution (pH 12) in E) top view and F) 3D.

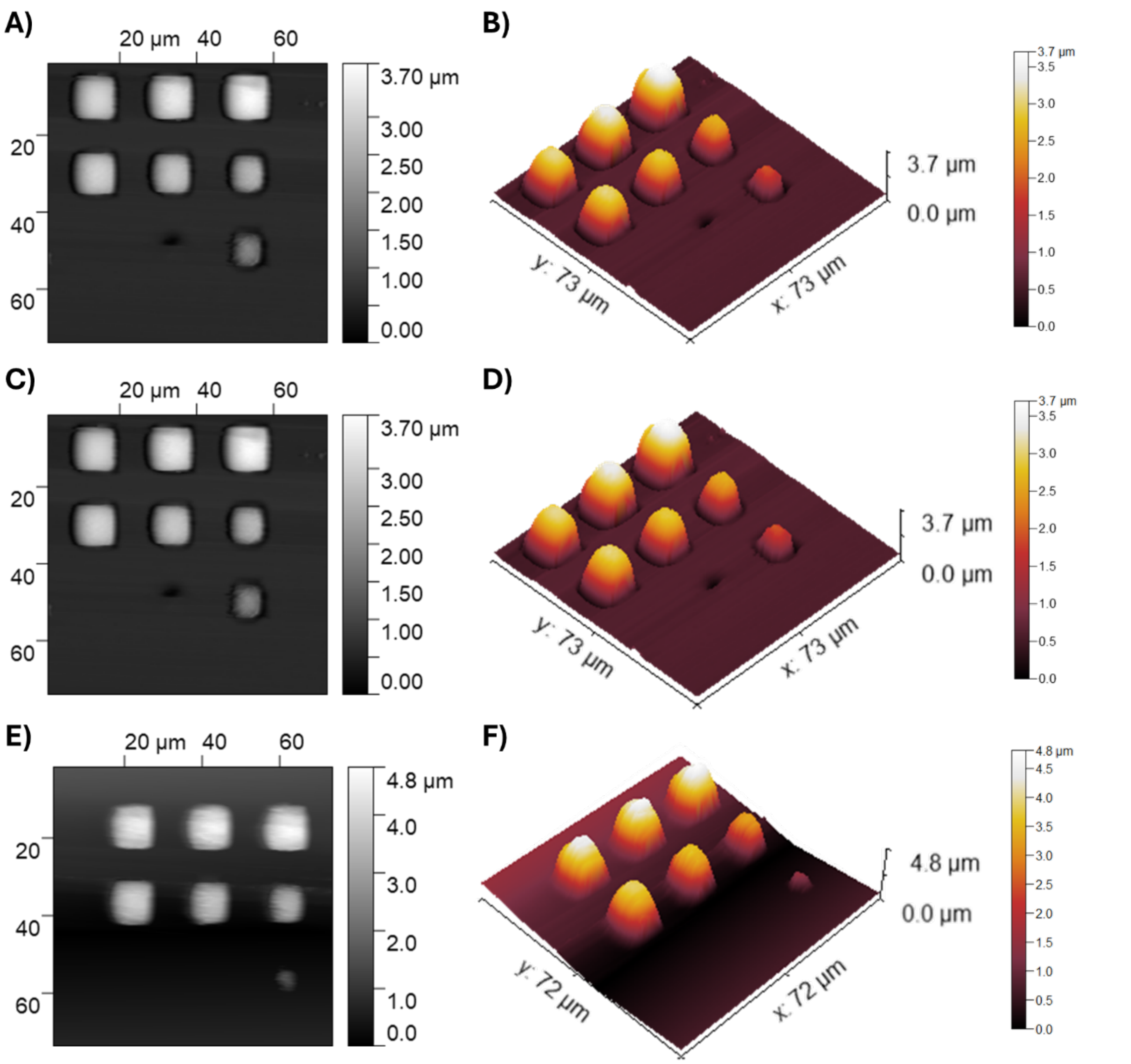

Figure S9. Topography obtained by AFM of p(AMPS) micro-cubes in hydrated state in HCl solution (pH 2.7) A) top view and B) 3D; in deionized water C) top view and D) 3D; in NaOH solution (pH 12) in E) top view and F) 3D.

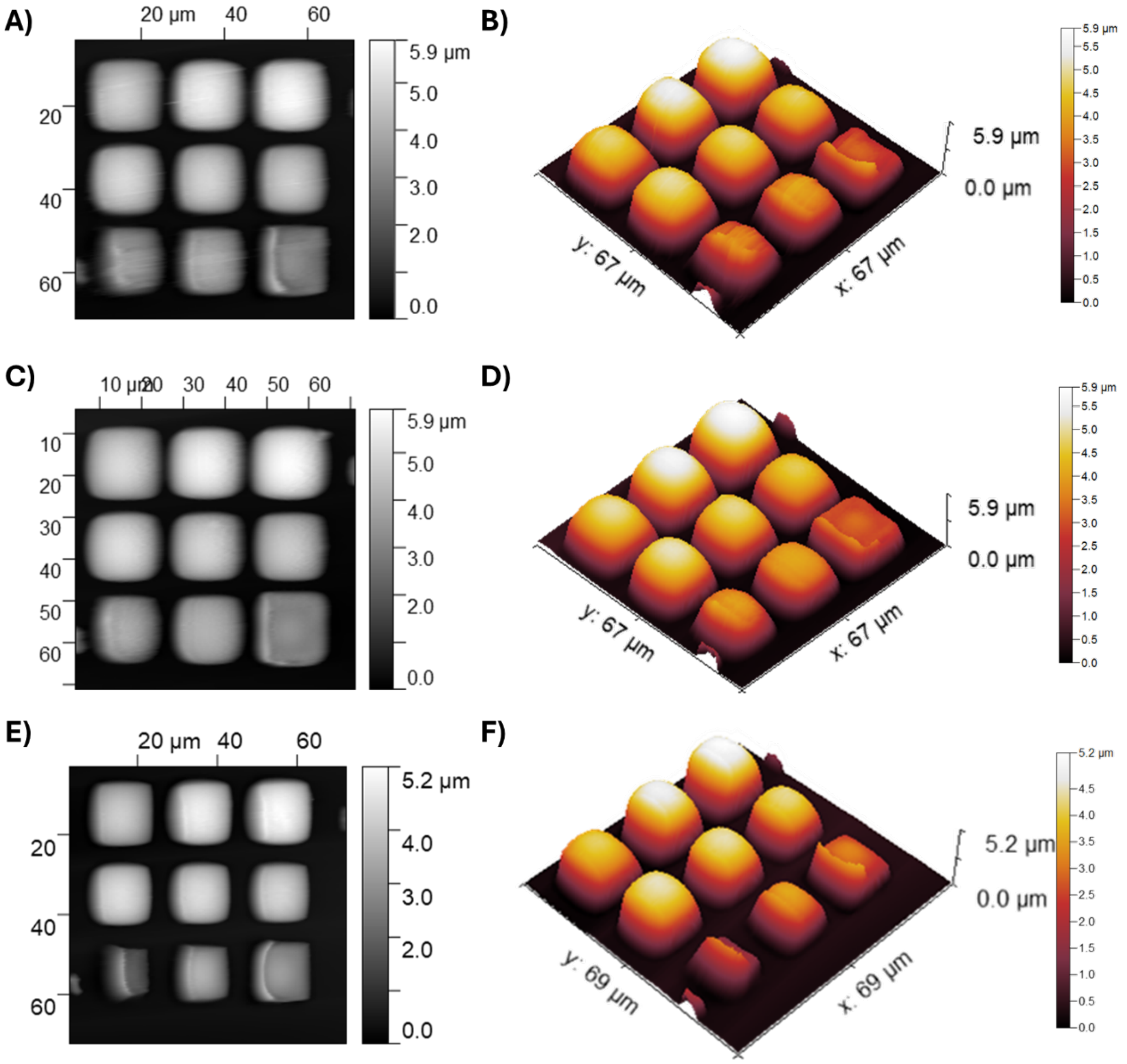

Figure S10. Topography obtained by AFM of p(APTAC) micro-cubes in their hydrated state in HCl solution (pH 2.7) A) top view and B) 3D; in deionized water C) top view and D) 3D; in NaOH solution (pH 12) in E) top view and F) 3D.

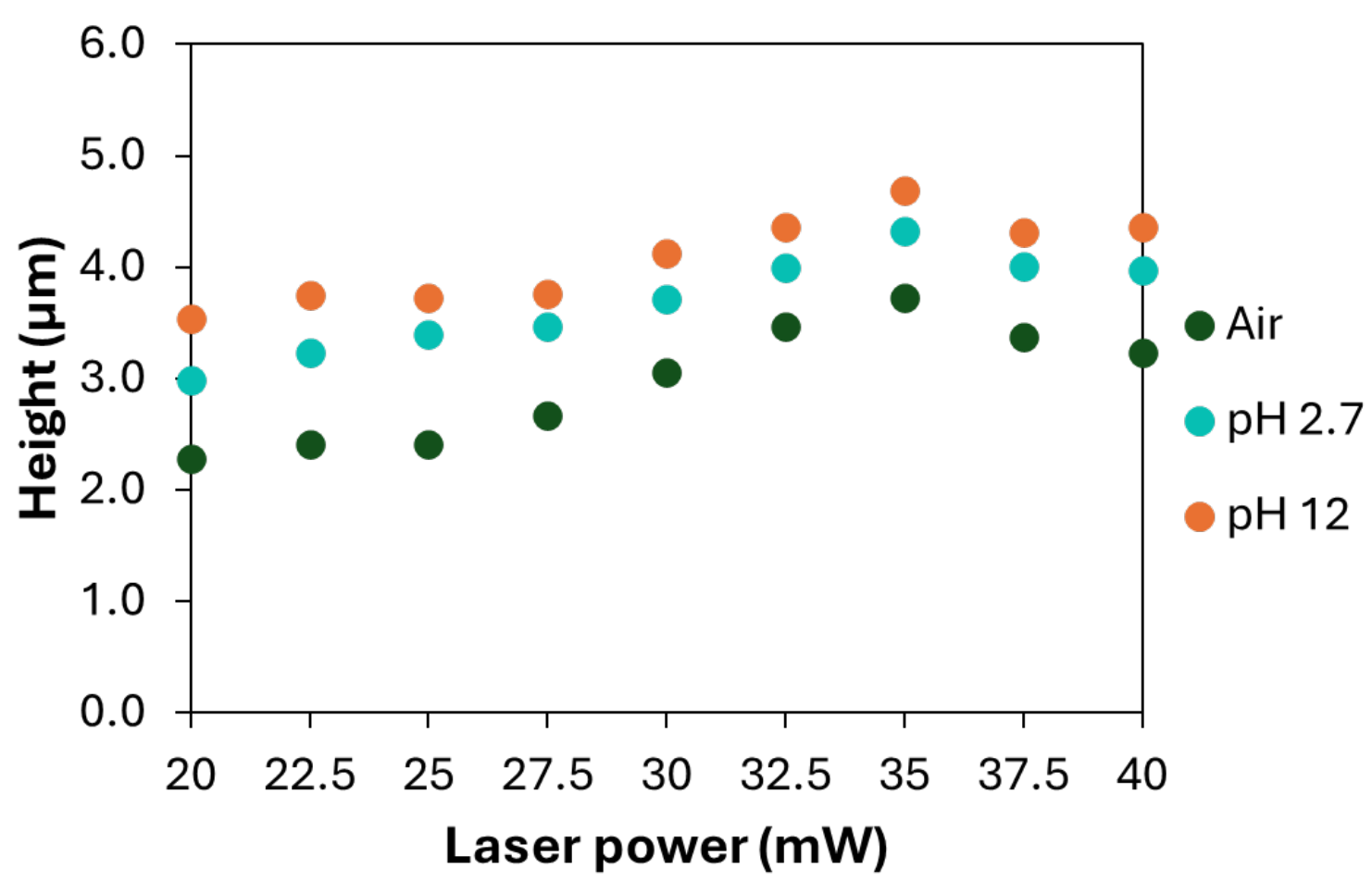

Figure S11. Effect of the laser power on the height of the p(AA) micro-cubes in the dry state, and hydrated state at pH 2.7 (HCl solution) and pH 12 (NaOH solution).

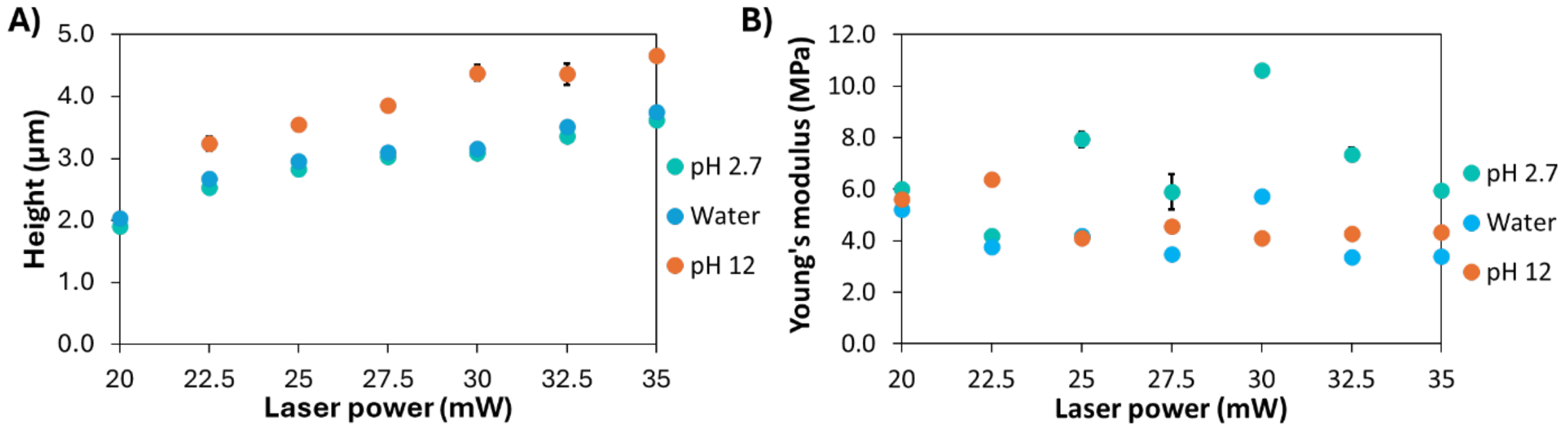

Figure S12. Effect of the laser power on A) the height and B) the Young's modulus of the p(AMPS) micro-cubes in the hydrated state at pH 2.7 (HCl solution), pH 12 (NaOH solution) and deionized water.

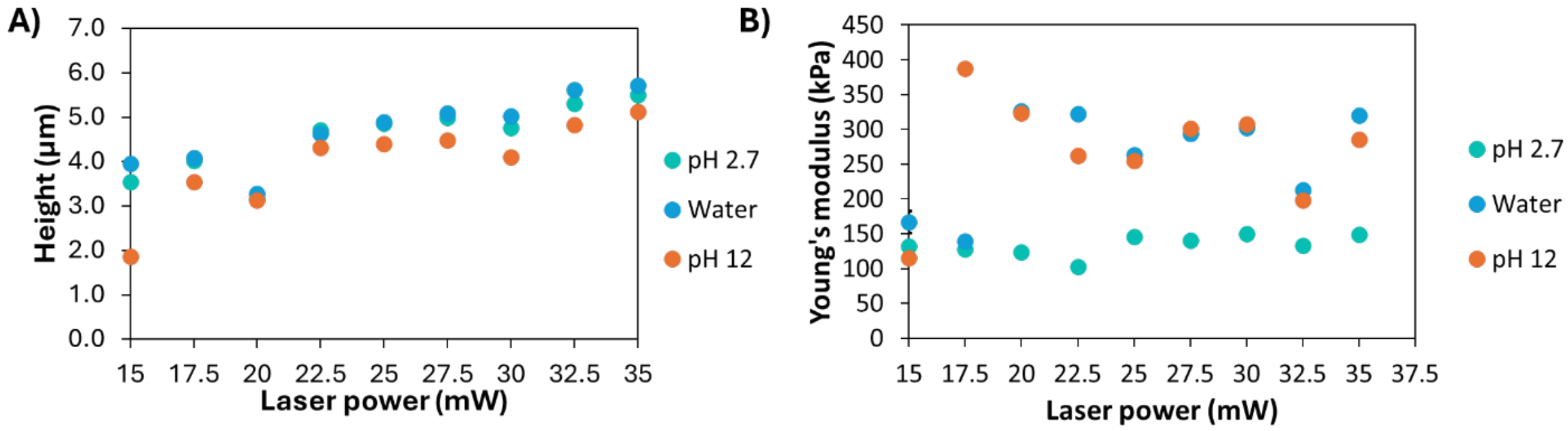

Figure S13. Effect of the laser power on A) the height and B) the Young's modulus of the p(APTAC) micro-cubes in the hydrated state at pH 2.7 (HCl solution), pH 12 (NaOH solution) and deionized water.

Fabrication of micro-cantilevers

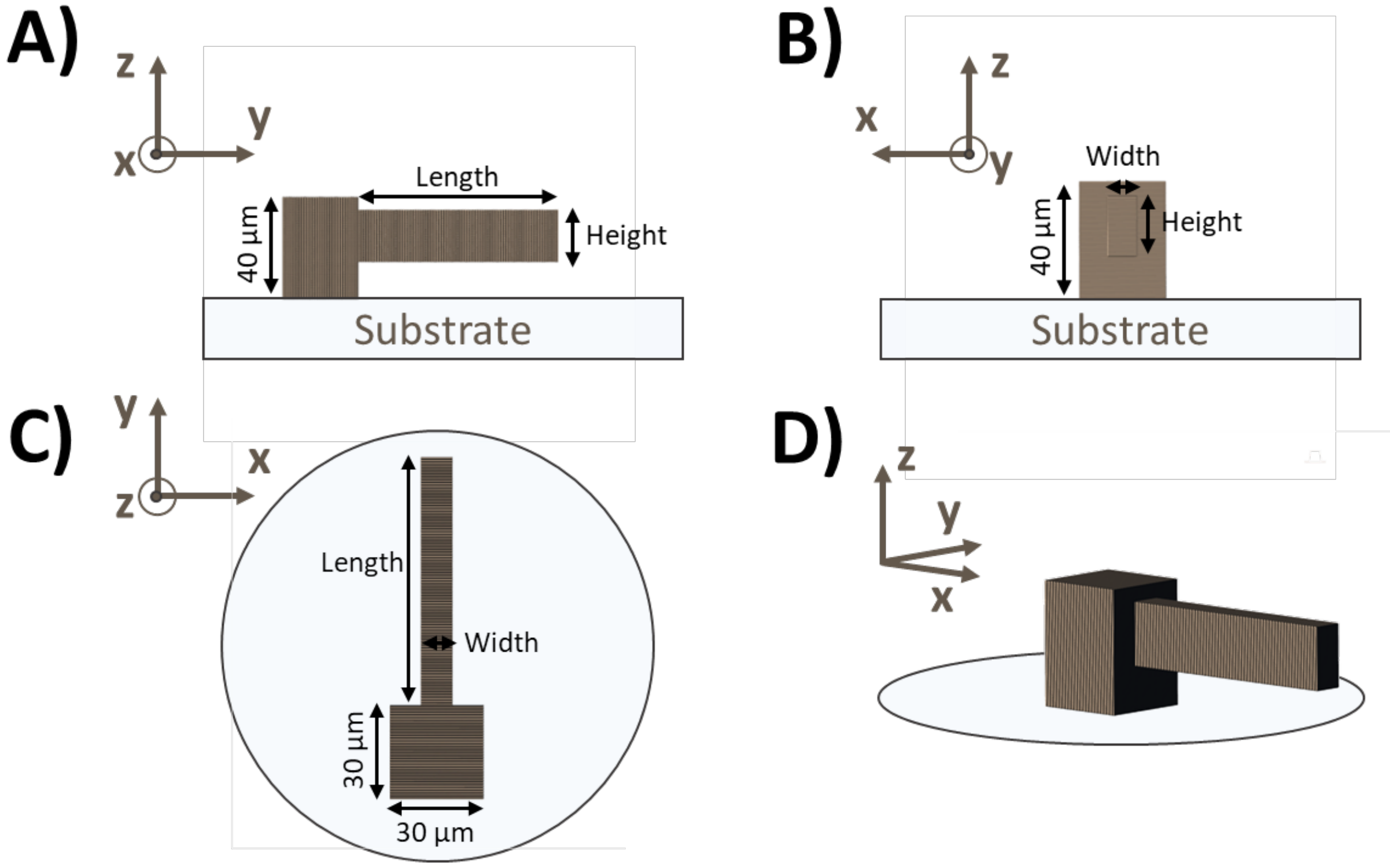

Figure S14. Design of the micro-cantilevers fabricated *via* 2PP in: A) Profile view, B) Frontal view, C) Top view, and D) and Three-quarters view.

Initially, micro-fabrication trials of the micro-cantilevers were done by scanning the laser power for a constant scanning speed of 10 mm s^{-1}, for a slicing and hatching distance of 0.3 µm. Such distances were chosen to preserve the robustness of the structures to self-support and withstand the post-fabrication washing and drying steps, while lowering their crosslinking density hence increasing their swelling and bending abilities. The high scanning speed was chosen to reduce fabrication times. If the micro-fabrication was not successful, the scanning speed was lowered, and the laser power was scanned again, until a set of successful micro-fabrication parameters was found. The success of micro-fabrication was assessed in post-fabrication steps, using optical microscopy.

Table S1. Parameters used for the fabrication of the macro-scale cantilever based on p(AA) using the Anycubic 3D printer Photon S.

Parameters \ Photoresist	p(AA)
Layer thickness (mm)	0.100
Exposure time (s)	20
Off-time (s)	1.00
Bottom exposure time (s)	35
Number of bottom layers	5
Z lift distance (mm)	5
Z lift speed (mm/s)	3
Z retract speed (mm/s)	3
Anti-alias	1

Multi-material fabrication

The fabrication of the multi-material structures was performed following a three-step fabrication process as described in the Experimental section. This fabrication process consisted of sequentially fabricating three distinct components of a gripper-like structure using three different photoresists: a base, a first microcantilever, and a second microcantilever. Each microcantilever was embedded 6 µm along the y-axis and 5 µm along the x-axis (measured from the edges) into the base, to ensure good interlayer adhesion (**Figure S15B**). This design promoted interpenetration of the two acrylate-based polymer networks and increased the local crosslinking density within the overlap region.

To prepare the initial sample, a silanized glass substrate was mounted onto the corresponding sample holder. The silanization treatment prevented the delamination of the structure upon swelling by promoting the formation of covalent bonds between the glass substrate and the microstructures. Once mounted, alignment marks were drawn on the substrate to correspond with the reference markers previously positioned on the sample holder. A pressure sensitive adhesive (PSA) well was then attached to the substrate and covered using a non-treated coverslip, before introducing the desired photoresist into the home-made cell by capillarity. The sample holder was flipped and a drop of oil (Zeiss Immersol 518F) was added in the center of the other face of the silanized slide. For each fabrication step, the sample was prepared following this procedure, and precise alignment of the structures was achieved by matching the

substrate markers with the corresponding reference markers on the sample holder (**Figure S15A**).

Following the preparation of the initial sample, the three-step fabrication method proceeds as follows. In the first step, the sample was prepared with photoresist n°1 as aforementioned to fabricate the base of the structure. Once the fabrication was completed, the sample was removed from the sample holder, subsequently washed to remove any unpolymerized photoresist, and dried under a nitrogen stream before proceeding to the 2nd fabrication step (**Figure S15C, (i)**). In the second step, the sample was prepared with photoresist n°2 to fabricate the first micro-cantilever of the structure. Partial embedding of the micro-cantilever within the base promoted interpenetration of the two polymer networks and increased the local crosslinking density at the interface. The sample was subsequently washed and dried as previously described, before proceeding to the 3rd fabrication step (**Figure S15C, (ii)**). In the third step, the sample was prepared with photoresist n°3 to fabricate the second micro-cantilever of the structure. As in the previous step, partial embedding of the microcantilever within the base structure promoted interpenetration of the polymer networks, enhancing interfacial adhesion between the two materials. The sample was subsequently washed and dried to obtain the final multi-material structure (**Figure S15C, (iii)**).

Following this three-step fabrication process, well-defined components of the structure were obtained after each fabrication step (**Figure S15D**).

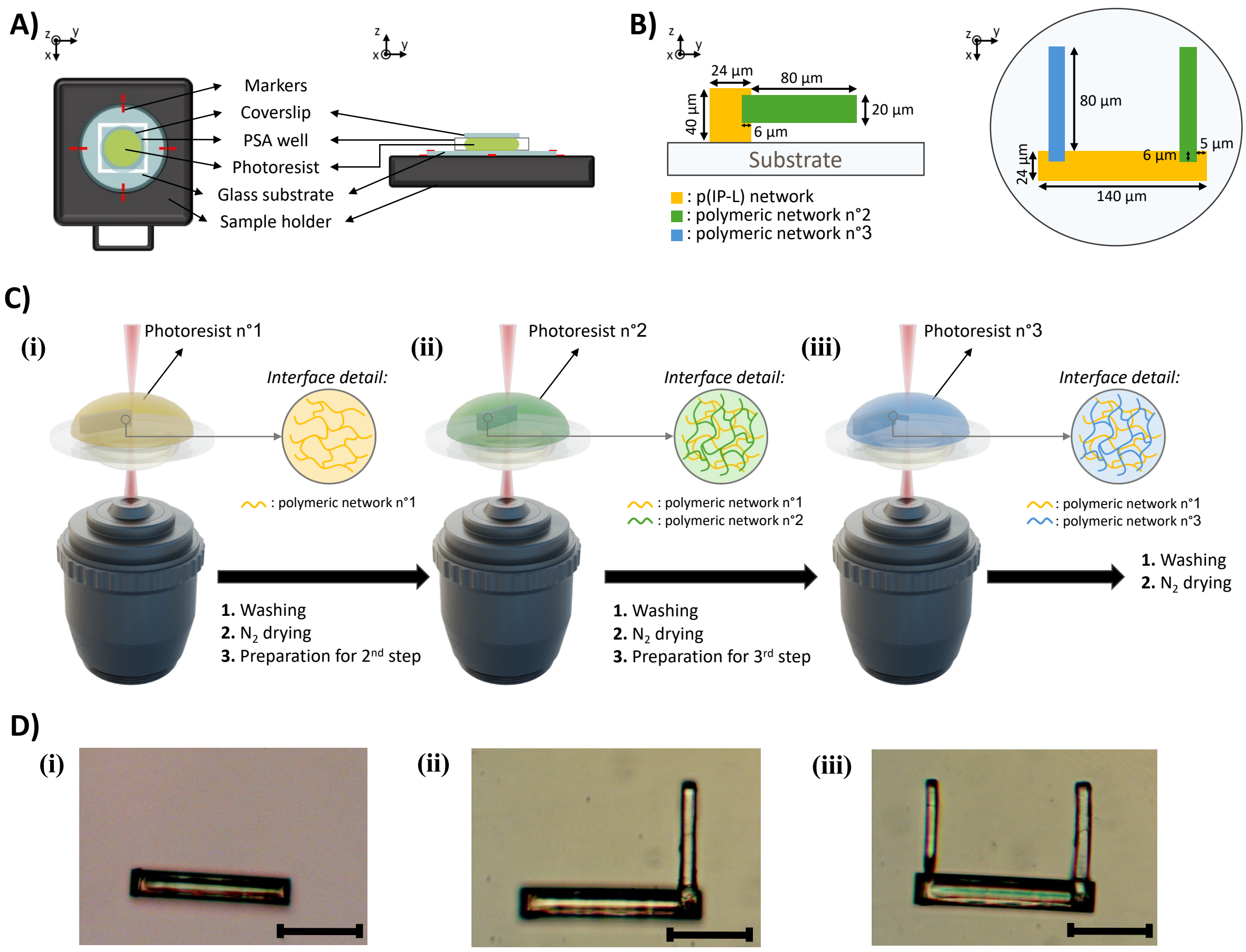

Figure S15. **Multi-material fabrication process. (A)** Schematic of the sample preparation showing the marker lines used to ensure consistent positioning during the successive fabrication steps, presented in top view (*Left*) and side view (*Right*). **(B)** Design of the multi-material microgripper fabricated *via* two-photon polymerization (2PP), shown in side view (*Left*) and top view (*Right*). **(C)** Schematic illustrating the three-step fabrication process of the multi-material structures. The base of the structure was fabricated in step one using photoresist n°1 **(i)**, followed by the fabrication of the first micro-cantilever in step two using photoresist n°2 **(ii)**, finally the second micro-cantilever was fabricated in step three using photoresist n°3, before yielding the final structure after washing and drying of the sample **(iii)**. **D)** Microscope images of the p(AA)\p(APTAC) multi-material structure after the first fabrication step **(i)**, after the second fabrication step **(ii)**, and after the third fabrication step **(iii)**. The scale bar represents 50 µm.

Electro-actuation of micro-cantilevers

Channels fabrication

The channels used for the electro-actuation experiments were designed using Blender 2.90®, sliced using the Photon Workshop V2.124 RC7® (Anycubic), and 3D printed on an Anycubic Photon Mono printer using the Standard White Resin (Anycubic) with the parameters listed in

Table S2. After fabrication, the channels were washed in an ethanol bath for 6 min and cured for 20 min at 405 nm, using an Anycubic Wash & Cure 1.

Table S2. Parameters used for the fabrication of the 3D printed channels using the Anycubic 3D printer Photon Mono.

Parameters \ Photoresist	Standard white resin
Layer thickness (mm)	0.05
Exposure time (s)	2.5
Off-time (s)	1.00
Bottom exposure time (s)	25
Number of bottom layers	5
Z lift distance (mm)	6
Z lift speed (mm/s)	4
Z retract speed (mm/s)	6
Anti-alias	1

Experimental setup

Electro-actuation experiments were carried out using 3D printed channels. These incorporated walls patterned with spikes, in order to prevent bubbles generated by water electrolysis from reaching the micro-cantilever structures (**Figure S16A** and **B**). The design of the 3D printed channel, the experimental set-up, and the measurement method used to characterize the electro-actuation of the micro-cantilevers are described in the figure below (**Figure S16**).

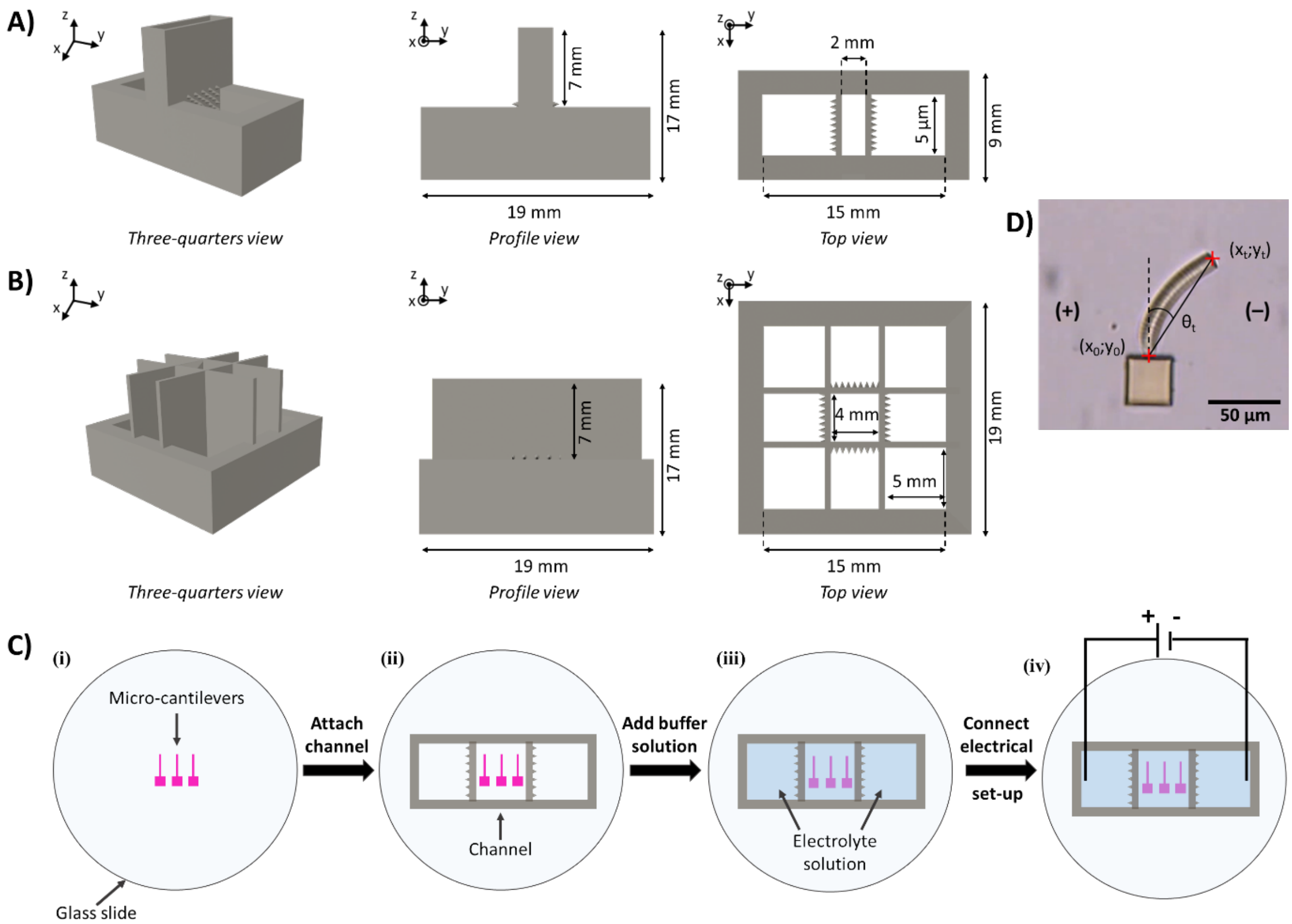

Figure S16. A) 3D design of the channel used for the electro-actuation of EAH microstructures using two electrodes, in different views. B) 3D design of the channel used for the electro-actuation of EAH microstructures using four electrodes, in different views. C) Experimental set-up used for the electro-actuation of EAH microstructures. Initially, the PSA well was removed from the glass substrate on which the structures were fabricated (i). A 3D printed channel was attached to the glass substrate using a commercially available epoxy glue (ii). The channel was then filled with the desired electrolyte solution (iii). Finally, the electrodes were immersed in the electrolyte solution and connected to a customized polarity switch controller, which was then connected to the power source. (iv). D) Image of a p(AA) micro-cantilever bending in response to an applied electric field and schematic of the measurement of its bending angle, $\boldsymbol{\theta_t}$.

Bending angle measurements

The bending angle was calculated by using the coordinates of the two endpoints of the cantilever (**Figure S16D**) as described in **Equation S1**. By convention, the bending angle was given a positive value when the cantilever bent towards the right, whereas a negative value was given when the cantilever bent towards the left.

$$\boldsymbol{\theta_t = tan^{-1}\left(\frac{|x_0 - x_t|}{|y_0 - y_t|}\right)} \qquad \textbf{(Eq. S1)}$$

where θ_t is bending angle recorded at the time t (in °), x_0 and y_0 are the horizontal and vertical coordinates of the first endpoint at which the cantilever is attached to the base (in pixels), and x_t and y_t are the horizontal and vertical coordinates of the second endpoint corresponding to the tip of the cantilever (in pixels).

The average bending angle of the cantilever over five polarity switch cycles was obtained by calculating the average of the bending angles when the bending angle plateaued as described in **Equation S2**. The average bending angle of the positive values was calculated by only taking the plateau bending angle values when the cantilever bent towards the right, whereas the bending angle of the negative values was calculated by only taking the plateau bending angle values when the cantilever bent towards the left.

$$\boldsymbol{\theta}_{\text{average}} = \frac{\sum_{t=1}^{N} |\theta_t|}{N} \quad \textbf{(Eq. S2)}$$

where $\theta_{average}$ is the averaged bending angle (in °), θ_t is the bending angle of the cantilever (in °) at a certain time t, and N is the number of recorded values.

Electro-actuation data

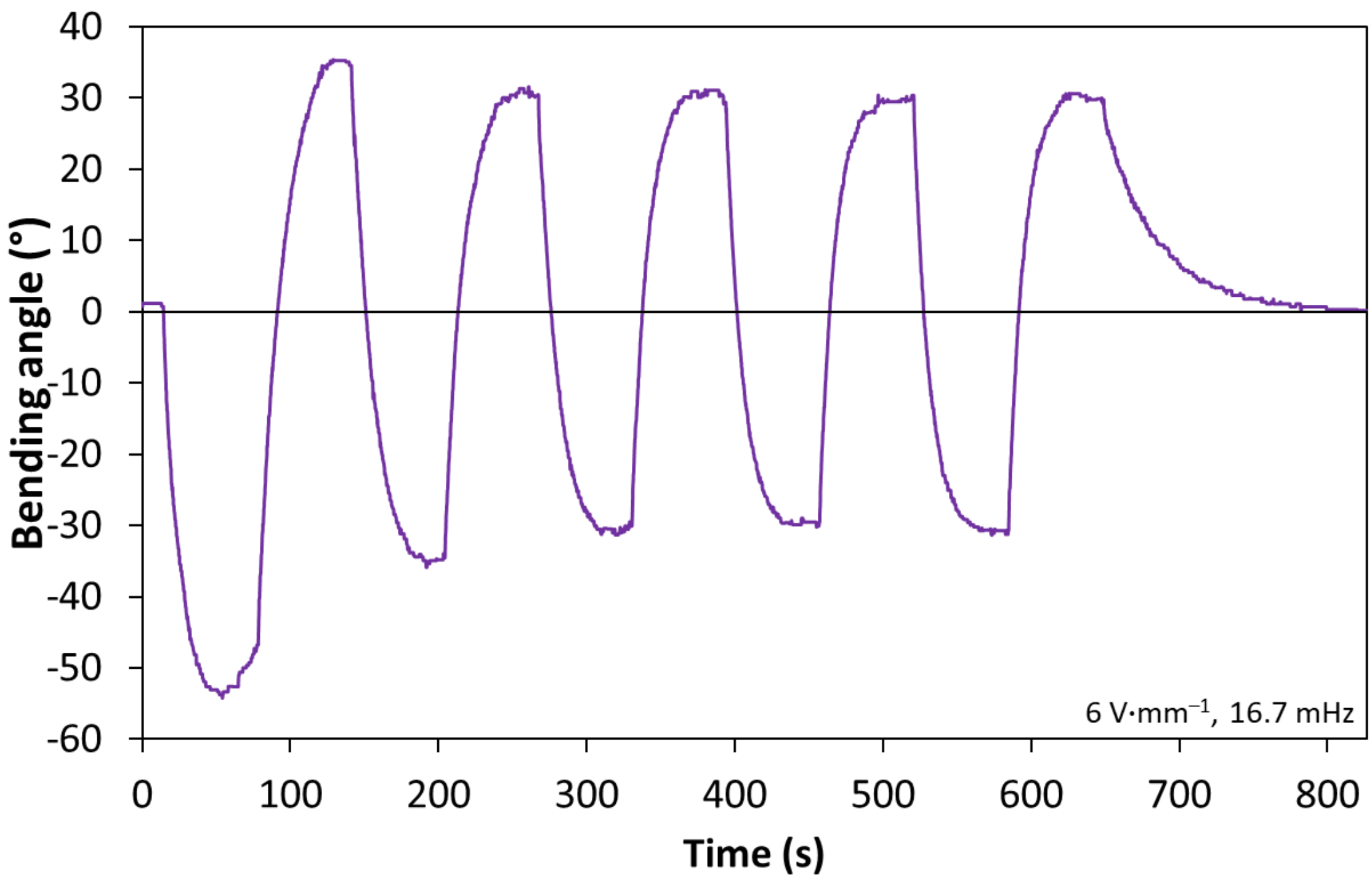

Figure S17. Electro-actuation of the macro-cantilever (8×1×2 mm^3) over 5 polarity switch cycles.

Table S3. Bending angle values of the p(AA) micro-cantilevers studied, depending on their length. The width and height of the cantilevers were kept constant at 10 µm and 20 µm, respectively. Electroactuation was performed in 50 mM phosphate buffer solution of pH 7.4, under an electric field of 4 V mm^{-1}.

Length [µm]	Positive [°]	Negative [°]	Overall [°]
50	13.2 ± 0.9	-9.0 ± 1.1	10.9 ± 2.3
60	14.3 ± 1.3	-9.8 ± 1.7	11.9 ± 2.7
70	16.6 ± 1.8	-11.5 ± 2.2	13.9 ± 3.2
80	19.2 ± 1.9	-16.3 ± 2.3	17.7 ± 2.6
90	21.6 ± 1.9	-15.3 ± 2.5	18.3 ± 3.8

Table S4. Bending angle values of the p(AA) micro-cantilevers studied, depending on their width. The length and height of the cantilevers were kept constant at 80 µm and 20 µm, respectively. Electroactuation was performed in 50 mM phosphate buffer solution of pH 7.4, under an electric field of 4 V mm^{-1}.

Width [µm]	Positive [°]	Negative [°]	Overall [°]
5	29.1 ± 1.2	-18.9 ± 1.7	24.5 ± 5.3
10	21.1 ± 0.6	-14.3 ± 0.6	18.0 ± 3.4
15	15.2 ± 0.7	-14.6 ± 0.8	15.0 ± 1.2
20	10.9 ± 0.9	-10.0 ± 0.6	10.5 ± 1.4
25	9.0 ± 0.7	-7.3 ± 0.5	8.3 ± 1.0
30	7.5 ± 0.5	-5.5 ± 0.6	6.6 ± 1.1

Table S5. Bending angle values of the p(AA) micro-cantilevers studied, depending on their height. The length and width of the cantilevers were kept constant at 80 µm and 10 µm, respectively. Electroactuation was performed in 50 mM phosphate buffer solution of pH 7.4, under an electric field of 4 V mm^{-1}.

Height [µm]	Positive [°]	Negative [°]	Overall [°]
10	11.7 ± 1.0	-9.1 ± 1.3	10.4 ± 1.6
15	17.4 ± 0.8	-15.7 ± 1.4	16.6 ± 1.4
20	17.4 ± 1.9	-15.9 ± 0.3	16.7 ± 1.6
25	19.0 ± 1.0	-16.2 ± 1.2	17.7± 1.8

Table S6. Bending angle values of the different micro-cantilevers studied, depending on the electric field used. The micro-cantilever dimensions were kept constant, at $80 \times 10 \times 20$ µm^3. Electroactuation was performed in 50 mM phosphate buffer solution of pH 7.4.

Electric field [V mm^{-1}]	p(APTAC)			p(AMPS)			p(AA)		
	Positive [°]	Negative [°]	Overall [°]	Positive [°]	Negative [°]	Overall [°]	Positive [°]	Negative [°]	Overall [°]
1	0.9 ± 0.4	-1.0 ± 0.3	1.0 ± 0.3	10.8 ± 1.4	-12.5 ± 2.5	11.5 ± 2.1	4.3 ± 0.6	-3.5 ± 0.5	3.9 ± 0.7
2	2.3 ± 0.8	-2.3 ± 0.9	2.3 ± 0.9	25.9 ± 5.6	-24.7 ± 3.2	25.3 ± 4.7	5.6 ± 1.0	-12.9 ± 1.1	9.2 ± 3.8
3	3.7 ± 1.6	-3.5 ± 1.3	3.6 ± 1.5	38.6 ± 6.3	-38.0 ± 3.6	38.3 ± 5.2	12.6 ± 1.6	-19.2 ± 1.2	15.6 ± 3.6
4	5.1 ± 1.8	-5.0 ± 1.9	5.0 ± 1.8	44.9 ± 5.2	-41.3 ± 4.0	43.1 ± 5.0	17 ± 3.0	-26.3 ± 2.3	21.1 ± 5.2
5	5.9 ± 2.4	-5.6 ± 1.8	5.7 ± 2.2	48.2 ± 5.4	-45.2 ± 3.4	46.7 ± 4.8	24.1 ± 2.9	-28.5 ± 1.3	26.3 ± 3.2
6	5.0 ± 2.3	-5.4 ± 1.6	5.2 ± 2.0	43.5 ± 8.0	-46.3 ± 7.3	44.9 ± 7.8	32.1 ± 2.7	-28.2 ± 2.0	30.2 ± 3.1

Table S7. Bending angle values of the different micro-cantilevers studied, depending on the electrolyte concentration used. The micro-cantilever dimensions were kept constant, at $80\times10\times20$ µm^3. Electroactuation was performed under an electric field of 4 V mm^{-1}.

Concentration [mM]	p(APTAC)			p(AMPS)			p(AA)		
	Positive [°]	Negative [°]	Overall [°]	Positive [°]	Negative [°]	Overall [°]	Positive [°]	Negative [°]	Overall [°]
25	3.9 ± 1.9	-3.7 ± 1.7	3.8 ± 1.8	39.6 ± 3.0	-39.1 ± 6.7	39.4 ± 5.2	19.8 ± 3.6	-13.1 ± 1.9	16.6 ± 4.5
50	5.1 ± 1.8	-5.0 ± 1.9	5.0 ± 1.8	38.7 ± 7.2	-42.8 ± 3.3	40.9 ± 5.9	20.4 ± 2.7	-15.3 ± 2.6	18.3 ± 3.7
75	5.4 ± 1.5	-5.7 ± 1.3	5.6 ± 1.4	40.5 ± 2.7	-37.2 ± 2.0	38.8 ± 2.9	19.7 ± 2.7	-19.2 ± 2.5	19.5 ± 2.6
100	5.5 ± 1.3	-5.7 ± 1.3	5.6 ± 1.4	41.5 ± 2.9	-38.1 ± 2.2	39.8 ± 3.1	19.4 ± 3.1	-20.9 ± 3.2	20.1 ± 3.2
125	5.7 ± 1.2	-5.6 ± 1.3	5.7 ± 1.2	41.4 ± 4.1	-32.9 ± 3.2	37.0 ± 5.6	20.7 ± 3.6	-22.8 ± 4.2	21.8 ± 4.1
150	5.1 ± 1.0	-5.8 ± 1.0	5.4 ± 1.1	32.8 ± 2.6	-31.1 ± 2.5	31.9 ± 2.7	16.8 ± 3.6	-21.7 ± 3.8	19.2 ± 4.4
175	5.4 ± 0.9	-5.3 ± 0.8	5.3 ± 0.9	29.2 ± 2.2	-27.5 ± 2.0	28.3 ± 2.3	17.4 ± 3.5	-24.8 ± 2.8	21.2 ± 4.8
200	4.6 ± 1.0	-5.1 ± 0.9	4.9 ± 1.0	25.3 ± 3.0	-24.5 ± 1.9	24.9 ± 2.5	14.8 ± 3.5	-24.6 ± 2.6	19.6 ± 5.8

Table S8. Bending angle values of the different micro-cantilevers studied, depending on the pH of the electrolyte concentration used. The micro-cantilever dimensions were kept constant, at 80×10×20 μm^3. Electroactuation was performed under an electric field of 4 V mm^{-1}.

pH	p(APTAC)			p(AMPS)			p(AA)		
	Positive [°]	Negative [°]	Overall [°]	Positive [°]	Negative [°]	Overall [°]	Positive [°]	Negative [°]	Overall [°]
3.2	11.2 ± 3.6	-11.1 ± 3.0	11.1 ± 3.3	25.2 ± 3.1	-18.8 ± 2.8	21.7 ± 4.3	0	0	0
7.4	5.1 ± 1.8	-4.9 ± 1.9	5.0 ± 1.8	41.3 ± 4.9	-44.1 ± 3.8	42.8 ± 4.6	22.3 ± 1.6	-14.9 ± 3.0	18.6 ± 4.4
10.6	5.0 ± 2.0	-7.2 ± 1.4	6.0 ± 2.1	39.2 ± 2.1	-38.6 ± 3.6	38.9 ± 3.0	18.6 ± 1.6	-13.8 ± 1.1	16.2 ± 2.7

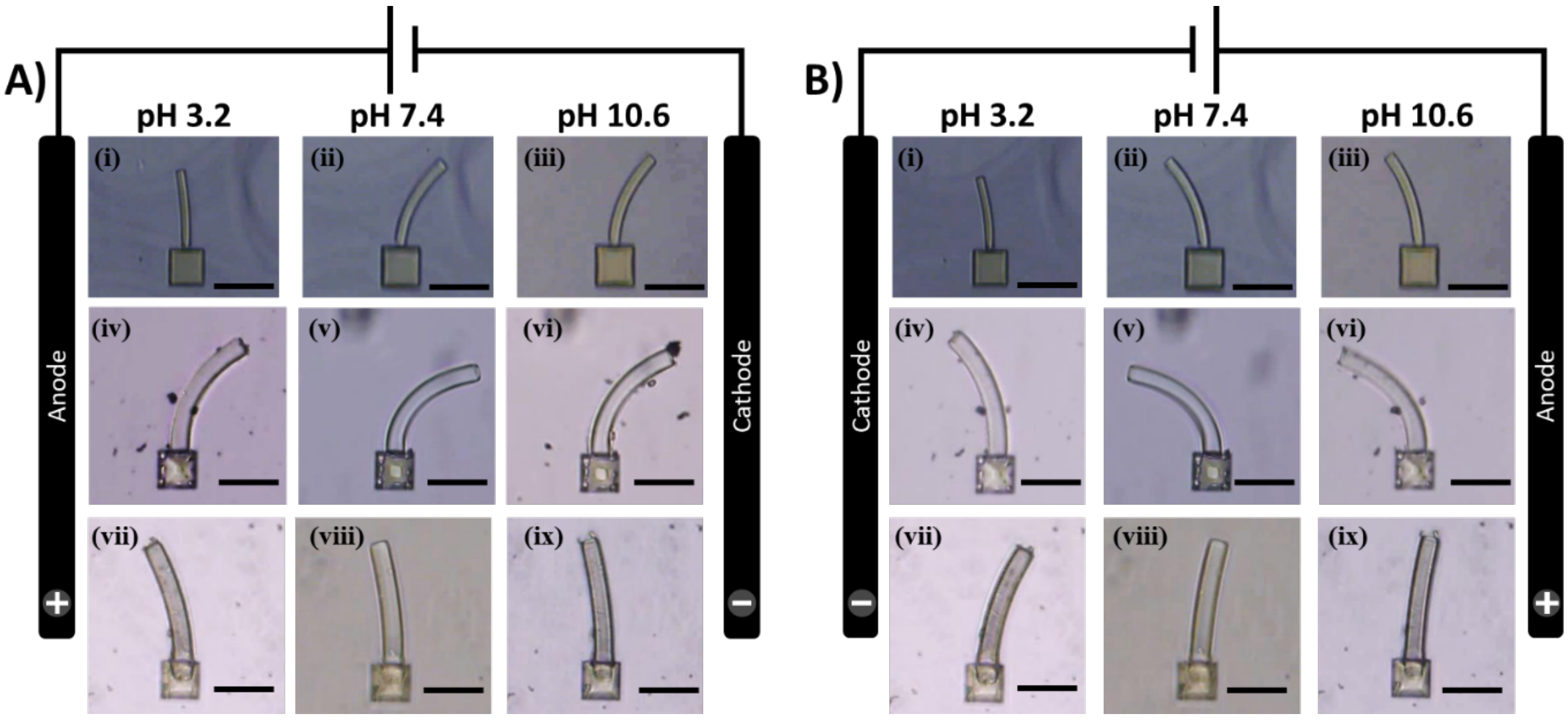

Figure S18. Microscopy images of the p(AA) (i-iii), the p(AMPS) (iv-vi), and the p(APTAC) (vii-ix) micro-cantilevers in electrolyte solutions of pH 3.2 (i, iv, vii), pH 7.4 (ii, v, viii), and pH 10.6 (iii, vi, ix) upon applying A) an electric field (4 V mm^{-1}) and B) upon reversing the direction of the electric field (4 V mm^{-1}). The scale bars represent 50 μm.

Quantitative cycle-life testing (> 100 cycles)

Quantitative cycle-life experiments were carried out at varying electric field strengths to determine the durability and repeatability of the actuation. This experiment was carried out for p(AMPS) micro-cantilevers (80×10×20 μm) fabricated using a laser power of 27.5 mW at 1.0 mm s^{-1}, over 12 months prior to this study. Electroactuation was performed in 50 mM phosphate buffer solution of pH 7.4, for electric fields of 1 V mm^{-1}, 2 V mm^{-1}, 3 V mm^{-1}, 4 V mm^{-1}, 5 V mm^{-1}, and 6 V mm^{-1}, respectively. Overall bending angle of 10.4 ± 1.9 °(n= 324) at 1 V mm^{-1}, 18.7 ± 1.5 °(n= 276) at 2 V mm^{-1}, 27.5 ± 2.4 °(n= 647) at 3 V mm^{-1}, 35.3 ± 2.6 °(n= 1368) at 4

V mm^{-1}, 40.1± 3.0 °(n= 1751) at 5 V mm^{-1}, and 46.2 ± 1.7 °(n= 3218) at 6 V mm^{-1}, respectively, were recorded for the first five polarity cycles. While no measurable deterioration in actuation performance was recorded after over 100 cycles at 1 V mm^{-1} and 2 V mm^{-1}, prolonged exposure to electric fields ≥4 V/mm, revealed a steady decrease in the actuation angle during cycling. Electrochemically generated gases at the electrodes under the applied electric field (≥4 V/mm) disrupt the local electric field distribution and promote water loss through evaporation, as evidenced by the observed decrease in electrolyte volume. The resulting increase in the ionic concentration of the electrolyte solution also contributes to the decrease in actuation performance, as supported by results in Figure 6D, which shows the effect of the concentration of the electrolyte solution on the bending angle of the p(AMPS) micro-cantilevers. Therefore, the electrolyte solution was replaced by a fresh one upon a noticeable change in the degree of actuation for the experiment carried out at 3 V mm^{-1} and above. Renewal of the electrolyte solution (marked in Figure S19) enables recovery of the initial actuation performance, with bending angle showing a negligible variation of the degree of actuation through at least 100 polarity switches. Increasing the electric field strength to ≥5 V/mm not only results in a gradual decline in actuation performance over time but can also compromise the reliability of automated actuation tracking (Figure S19E). This effect becomes apparent for 5 V/mm after approximately 15 actuation cycles (Figure S19E), when the generation of large number of gas bubbles interferes with the accurate tracking of actuator motion. Nevertheless, renewal of the electrolyte solution (marked in Figure S19C-F) restores the actuator's performance to that observed at the beginning of the cycle.

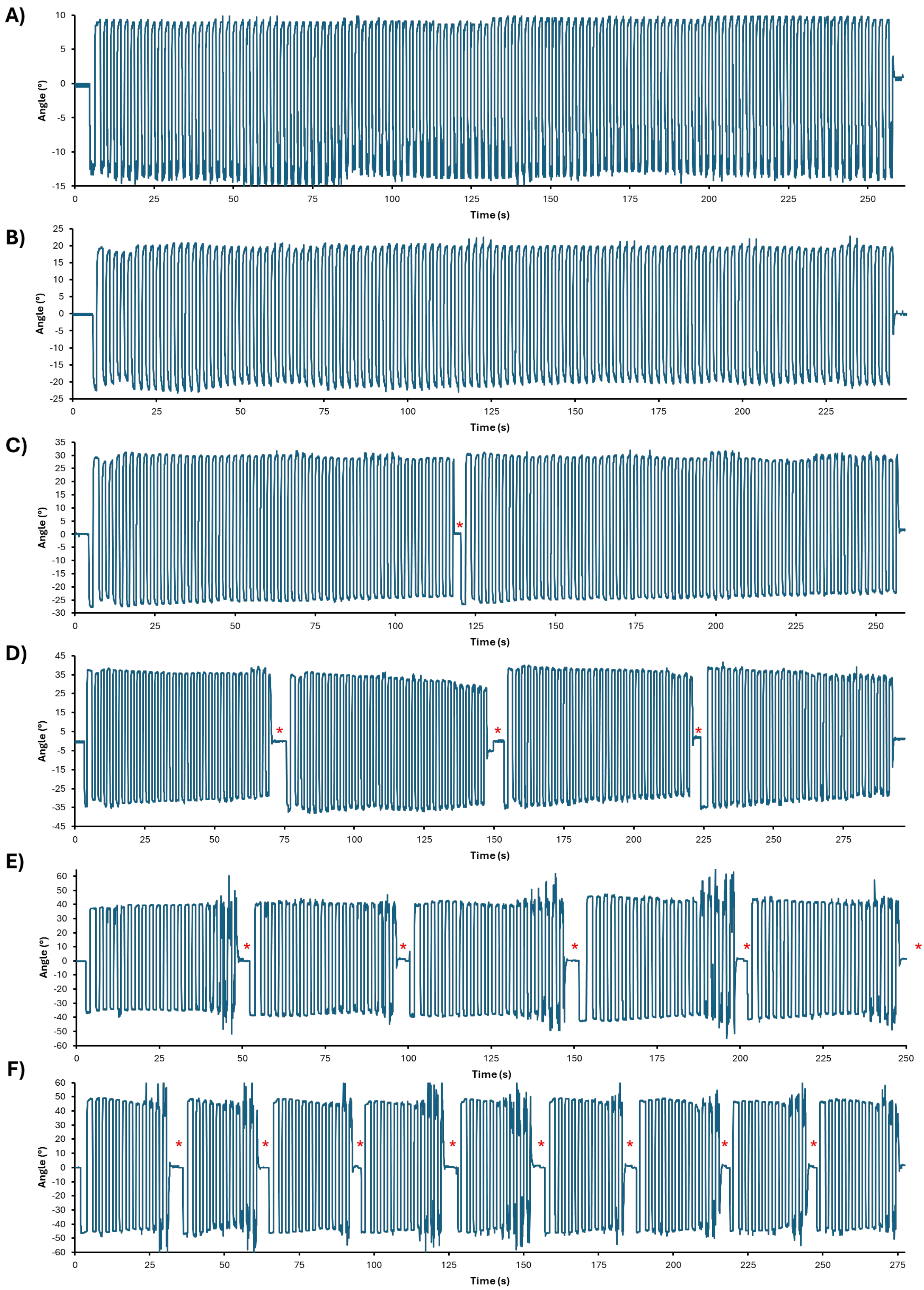

Figure S19. Measurements of the bending angle of the p(AMPS) micro-cantilevers ($80\times10\times20$ μm^3) fabricated using a laser power of 27.5 mW at 1.0 mm s^{-1} over at least 100 polarity cycles upon application of an electric field of A) 1 V mm^{-1}, B) 2 V mm^{-1}, C) 3 V mm^{-1}, D) 4 V mm^{-1}, E) 5 V mm^{-1}, F) 6 V mm^{-1}. These actuation cycles are performed on cantilever samples >12 months after micro-fabrication. * denotes renewal of the electrolyte solution.

Ion mobility

Table S9. Properties of mobile ions available in the different electrolyte solutions tested.

Ion	Mobility, μ, [10^{-8} $m^2 \cdot s^{-1} \cdot V^{-1}$]	Ionic radii [nm]	Ionic conductivity [$S \cdot cm^2 \cdot mol^{-1}$]	Drift velocity [in $\mu m \cdot s^{-1}$]					
				1 V mm^{-1}	2 V mm^{-1}	3 V mm^{-1}	4 V mm^{-1}	5 V mm^{-1}	6 V mm^{-1}
H^+	36.23	--	349.65	362.3	724.6	1086.9	1449	1811.5	2174
Na^+	5.19	0.102	50.1	51.9	103.8	155.7	207.6	259.5	311.4
K^+	7.62	0.138	73.5	76.2	152.4	228.6	304.8	381.0	457.2
HO^-	20.64	0.176	198	206.4	412.8	619.2	825.6	1032.0	1238
Cl^-	7.91	0.181	76.3	79.1	158.2	237.3	316.4	395.5	474.6

* The mobility values were presented by Varcoe et al.[1], the ionic conductivities were presented by Paul et al.[2]

The time required for the ions to travel a distance can be described as follows:

$$t_{ion} = \frac{d}{v_{ion}} \quad \textbf{(Eq. S3)}$$

Where t is the migration time of the counterion (in s), d is the distance travelled by the thickness of the hydrogel (in μm), v_{ion} is the drift velocity of the counterion (in μm s^{-1}).

Table S10. Migration time of the ions in the electrolyte solution to reach the nearest interface of the micro-cantilever (80×10×20 μm^3) across a 10 mm electrode gap, for different electric field values.

Ion	Migration time of the counterion [s]					
	1 V mm^{-1}	2 V mm^{-1}	3 V mm^{-1}	4 V mm^{-1}	5 V mm^{-1}	6 V mm^{-1}
H^+	13.8	6.9	4.6	3.4	2.8	2.3
Na^+	96.2	48.1	32.1	24.1	19.2	16.0
K^+	65.6	32.8	21.9	16.4	13.1	10.9
HO^-	24.2	12.1	8.1	6.1	4.8	4.0

Table S11. Migration time of the counterions in a micro-cantilever (80×10×20 μm^3) for different electric field values.

Ion	Migration time of the counterion [ms]					
	1 V mm^{-1}	2 V mm^{-1}	3 V mm^{-1}	4 V mm^{-1}	5 V mm^{-1}	6 V mm^{-1}
H^+	27.6	13.8	9.2	6.9	5.5	4.6
Na^+	192.7	96.3	64.2	48.2	38.5	32.1
K^+	136.1	65.6	43.7	32.8	26.2	21.9
HO^-	50.5	24.2	16.1	12.1	9.7	8.1
Cl^-	126.4	63.2	42.1	31.6	25.3	21.1

3D design of microstructures

The micro-scale cantilever structures were generated using MATLAB codes created by co-author Dr. Luke Dowling. The other designs, such as Frangipani flower, Hallgrímskirkja church, Owl, Babylon tower, Rose, Venus of Melos, and Water lily were adapted from files licensed under the Creative Commons - Attribution license, and available for download under the links below:

- Frangipani flower: by citarox licensed under the Creative Commons - Attribution license, and https://www.thingiverse.com/thing:2374610
- Hallgrímskirkja church: by utechlab licensed under the Creative Commons - Attribution license, and available at https://www.thingiverse.com/thing:1389710
- Owl: by BrOncO, is licensed under Creative Commons – Attribution, and is available at https://www.thingiverse.com/thing:693371
- Babylon Tower: by Iraq3DP licensed under the Creative Commons - Attribution license and available at https://www.thingiverse.com/thing:2874625
- Rose: by marsmathofficial licensed under the Creative Commons - Attribution - Non-Commercial and available at https://www.thingiverse.com/thing:3609748
- Venus of Melos: by smkmuseum, licensed under the Creative Commons - Public Domain Dedication license and available at https://www.thingiverse.com/thing:1892710
- Water lily: by guppyk licensed under the Creative Commons - Attribution license, and available at https://www.thingiverse.com/thing:4364395

The objective lens used in Figure 1 is a modification of the original design from Thorfynn (https://grabcad.com/library/microscope-objective-from-zeiss-1) which is available at https://www.thingiverse.com/thing:7246827 and is licensed under the Creative Commons – Attribution license.

Modification of aforementioned designs were carried out using a computer-aided design software (CAD), such as Blender 2.90®, or the DeScribe software, before being digitally sliced using the DeScribe software. All other 3D structures were designed and created in Blender 2.90® before being imported in the DeScribe software.

Videos

Movie S1. Micro-scale p(AA), p(AMPS), and p(APTAC) micro-cantilevers

Electro-actuation of the different micro-cantilevers of dimensions 80×10×20 μm^3 in a 3D printed holder filled with an electrolyte solution (50 mM phosphate buffer solution of pH 7.4), subjected to an electric field strength of 6 V mm^{-1} and reversing the polarity of the electrodes every second (1 Hz). The first part (0 s – 9 s) and the second part (10 s – 18 s) of the movie corresponds to the electro-actuation of the p(AA) micro-cantilevers and the p(AMPS) micro-cantilevers, respectively. When the electric field was applied, the cantilevers bent towards the cathode (-). Upon reversing the direction of the electric field, the cantilevers reversed their direction of bending towards the new location of the cathode (-). The third part (19 s – 29 s) of the movie corresponds to the electro-actuation of the p(APTAC) micro-cantilevers. When the electric field was applied, the cantilevers bent towards the anode (+). Upon reversing the direction of the electric field, the cantilevers reversed their direction of bending towards the new location of the anode (+).

Movie S2. Electro-actuation of the p(AA) macro-cantilever

Electro-actuation of the macro-cantilever of dimensions 8×1×2 mm^3 in a 3D printed holder filled with an electrolyte solution (50 mM phosphate buffer solution of pH 7.4), subjected to an electric field strength of 6 V mm^{-1}, with the polarity of the electrodes being changed with different frequencies. The first part (0 s – 34 s) of the movie corresponds to the polarity of the electrodes being switched every 1 s (1 Hz frequency). Upon application of the electric field the cantilevers bent towards the cathode (-). Upon reversing the direction of the electric field, the cantilevers reversed their direction of bending towards the new location of the cathode (-). The second part (35 s – 87 s) of the movie corresponds to the polarity being reversed with a 16.7

mHz frequency, demonstrating that the cantilever reaches a plateau when given enough time before reversing the polarity.

Movie S3. Electro-actuation of p(AA) micro-cantilevers of varied geometries

Electro-actuation of p(AA) micro-cantilevers of varied dimensions in a 3D printed holder filled with an electrolyte solution (50 mM phosphate buffer solution of pH 7.4), subjected to an electric field of 4 V mm^{-1} at 1 Hz. The first part (0s – 8 s) of the movie shows the electro-actuation of p(AA) microcantilevers of varied length, while keeping their width and height at 10 μm and 20 μm, respectively. The second part (8 s – 19 s) of the movie shows the electro-actuation of p(AA) microcantilevers of varied width, while keeping their length and height at 80 μm and 20 μm, respectively. The third part (19 s – 29 s) of the movie shows the electro-actuation of p(AA) microcantilevers of varied height, while keeping their length and width at 80 μm and 10 μm, respectively. The dimensions of the micro-cantilevers of the varied geometrical parameters are indicated below each one.

Movie S4. Electro-actuation of micro-cantilevers under different electric field strengths

Electro-actuation of the different micro-cantilevers of dimensions 80×10×20 μm^3 in a 3D printed holder filled with an electrolyte solution (50 mM phosphate buffer solution of pH 7.4), subjected to different electric field strengths at 1 Hz. The first part (0 s – 54 s) of the movie corresponds to the electro-actuation of the p(AA) micro-cantilevers under different electric field strength, the second part (54 s – 103 s) shows the electro-actuation of the p(AMPS) micro-cantilevers under different electric field strength, and finally, the third part (104 s – 159 s) shows the electro-actuation of the p(APTAC) micro-cantilevers under different electric field strength.

Movie S5. Electro-actuation of micro-cantilevers at different electrolyte concentrations

Electro-actuation of the different micro-cantilevers of dimensions 80×10×20 μm^3 in a 3D printed holder filled with an electrolyte solution of the desired concentration (phosphate buffer solution of pH 7.4), subjected to an electric field strength of 4 V mm^{-1} at 1 Hz. The first part (0 s – 74 s) of the movie corresponds to the electro-actuation of the p(AA) micro-cantilevers in phosphate buffer solutions of pH 7.4 at different concentrations, the second part (75 s – 144 s) corresponds to the p(AMPS) micro-cantilevers, and finally, the third part (145 s – 217 s) to the p(APTAC) micro-cantilevers.

Movie S6. Electro-actuation of multi-material structures

Electro-actuation of the different multi-material structures in a 3D printed holder filled with an electrolyte solution (50 mM phosphate buffer solution of pH 7.4), subjected to an electric field strength of 4 V mm^{-1} at 1 Hz. The first part (0 s – 26 s) of the movie corresponds to the electro-actuation of the p(AMPS)/p(APTAC) structures in electrolyte solutions of different pHs, the second part (26 s – 64 s) corresponds to the p(AMPS)/p(AA) structures and finally, the third part (64 s – 99 s) to the p(AA)/p(APTAC) structures.

Movie S7. Electro-actuation of 4D actuators

Electro-actuation of the different 4D actuator structures in a 3D printed holder filled with an electrolyte solution (50 mM phosphate buffer solution of pH 7.4), subjected to an electric field strength of 4 V mm^{-1} at 1 Hz. The first part (0 s – 10 s) of the movie corresponds to the electro-actuation of the p(AMPS) twisting pillar, the second part (10 s – 33 s) of the movie corresponds to the electro-actuation of the p(AMPS) steerable pillar.

References

[1] J. R. Varcoe, P. Atanassov, D. R. Dekel, A. M. Herring, M. A. Hickner, P. A. Kohl, A. R. Kucernak, W. E. Mustain, K. Nijmeijer, K. Scott, T. Xu, L. Zhuang, *Energy Environ. Sci.* **2014**, *7*, 3135.

[2] S. Paul, K. S. Choi, D. J. Lee, P. Sudhagar, Y. S. Kang, *Electrochim. Acta* **2012**, *78*, 649.
